# Phylogenomics resolves the higher-level phylogeny of herbivorous eriophyoid mites (Acariformes: Eriophyoidea)

**DOI:** 10.1186/s12915-024-01870-9

**Published:** 2024-03-22

**Authors:** Qi Zhang, Yi-Wen Lu, Xin-Yu Liu, Ye Li, Wei-Nan Gao, Jing-Tao Sun, Xiao-Yue Hong, Renfu Shao, Xiao-Feng Xue

**Affiliations:** 1 Department of Entomology, Nanjing Agricultural University, Nanjing, 210095 Jiangsu China; 2 Centre for Bioinnovation, School of Science, Technology and Engineering, University of the Sunshine Coast, Sippy Downs, Queensland, 4556 Australia

**Keywords:** Divergence time, Eriophyoid mites, Gene order, Higher-level phylogeny, Mitochondrial genomes, Synteny

## Abstract

**Background:**

Eriophyoid mites (Eriophyoidea) are among the largest groups in the Acariformes; they are strictly phytophagous. The higher-level phylogeny of eriophyoid mites, however, remains unresolved due to the limited number of available morphological characters—some of them are homoplastic. Nevertheless, the eriophyoid mites sequenced to date showed highly variable mitochondrial (mt) gene orders, which could potentially be useful for resolving the higher-level phylogenetic relationships.

**Results:**

Here, we sequenced and compared the complete mt genomes of 153 eriophyoid mite species, which showed 54 patterns of rearranged mt gene orders relative to that of the hypothetical ancestor of arthropods. The shared derived mt gene clusters support the monophyly of eriophyoid mites (Eriophyoidea) as a whole and the monophylies of six clades within Eriophyoidea. These monophyletic groups and their relationships were largely supported in the phylogenetic trees inferred from mt genome sequences as well. Our molecular dating results showed that Eriophyoidea originated in the Triassic and diversified in the Cretaceous, coinciding with the diversification of angiosperms.

**Conclusions:**

This study reveals multiple molecular synapomorphies (i.e. shared derived mt gene clusters) at different levels (i.e. family, subfamily or tribe level) from the complete mt genomes of 153 eriophyoid mite species. We demonstrated the use of derived mt gene clusters in unveiling the higher-level phylogeny of eriophyoid mites, and underlines the origin of these mites and their co-diversification with angiosperms.

**Supplementary Information:**

The online version contains supplementary material available at 10.1186/s12915-024-01870-9.

## Background

Eriophyoid mites are in the highly speciose superfamily Eriophyoidea in the Acariformes, including more than 5000 taxonomically accepted species [1, 2]. They exhibit an uneven worldwide distribution, with most named species in temperate regions [3]. Eriophyoid mites are also among the smallest of terrestrial arthropods (averaging 200 μm in length) [4] and bear only two pairs of legs (known as “four-legged mites”). They are strictly phytophagous and have intricate relationships with host plants by making galls and blisters (thus known as “gall mites”) or living as vagrants on leaf surfaces; some of these species are pests and can cause massive economic losses in agriculture and forestry [5].

There has been a long-standing effort in erecting the classification system of eriophyoid mites. Historically, six morphology-based systems have been erected for the Eriophyoidea [4, 6–10]. The revised system of Amrine et al. [4] is widely used today—Eriophyoidea was divided into three families: Phytoptidae (ca. 160 species), Eriophyidae (ca. 3790 species) and Diptilomiopidae (ca. 450 species) [1, 4]. These systems are all morphology based and should be tested with other lines of evidence. Eriophyoid mites have several different morphological characters when compared with other mites, including fusiform or vermiform body shape, two pairs of legs, reduced setae on the opisthosoma and legs and a ringed opisthosoma [4]. These morphological characters were used unpolarized (plesiomorphic versus apomorphic) and repeatedly in taxonomic studies at different levels (subfamilial, tribal, or generic) [4]. Some of the morphological characters used to establish clades within Eriophyoidea were suggested as homoplastic due to convergent evolution [11]. Thus, these classification systems may not reflect the phylogenetic relationships of eriophyoid mites [4, 12–14]. Some previous molecular studies, using sequences of a small number of nuclear and mitochondrial gene segments of 10 to over 500 named species, further suggested the non-monophyly of all three families, and most subfamilies, tribes and genera of Eriophyoidea [11, 15–17].

The typical arthropod mitochondrial (mt) genome is circular, encoding 37 genes [18]. Phylogenomic studies often used nucleotide or amino acid sequences of mt genomes to resolve controversial relationships at different taxonomic levels of insects [19–25] and arachnids [26–30]. Changes in mt gene orders have also been explored for resolving higher-level phylogenies in arthropods [31–34]. For most arthropods the mt gene order is very conserved [21]. However, for arachnids, especially the Acariformes mites, the sequenced mt genomes to date indicate much more changes in mt gene orders [29, 30]. Our previous studies showed highly rearranged mt gene orders in four eriophyoid mite species [35, 36]. Given the scale of eriophyoid species diversity, there could be more changes in mt gene order among eriophyoid species. Eriophyoid mites thus represent an interesting lineage for which variation in mt gene orders may assist the resolution of their phylogenetic relationships.

Here, we compiled a dataset including the complete mt genomes of 153 eriophyoid mite species, of which 148 were newly sequenced in this study. Our dataset covers all three families (i.e. Phytoptidae, Diptilomiopidae and Eriophyidae), eight subfamilies, 13 tribes and 48 genera. We determined not only the extent of rearranged mt genomes in eriophyoid mites, but also their use for resolving the higher-level phylogeny of eriophyoid mites. We further examined the relationships between the extent of changed mt gene orders and the evolutionary rates in the arachnid lineages, and accounted for the evolutionary trajectory of eriophyoid mites.

## Results

### Highly rearranged mt gene orders in eriophyoid mites

We obtained the complete mt genomes of 153 putative species of eriophyoid mites, of which 148 were sequenced in the current study and five were sequenced previously (Additional file 1: Table S1; [35–37]). These eriophyoid species encompass three families, as well as eight subfamilies, 13 tribes and 48 genera (Additional file 1: Table S1). All obtained complete mt genomes are circular, encoding 13 protein-coding genes (PCGs), two rRNA genes and 22 tRNA genes. The mt genomes of 153 eriophyoid mite species show highly rearranged gene orders that involve genes for tRNAs, rRNAs and proteins (Additional file 2: Fig. S1), when compared with the hypothetical ancestral mt gene arrangement of arthropods [38]. These rearranged mt genomes show 54 different patterns (Patterns 1–54) which were manually compared and counted. These patterns were not found previously in any other arthropods (Additional file 2: Fig. S1). The 126 species of Eriophyidae have 38 different patterns of mt gene arrangement, the 22 species of Diptilomiopidae have 14 patterns and the five species of Phytoptidae have three patterns. Pattern 46 is widely distributed in nearly half (45.1%, 69/153) of eriophyoid species (Additional file 2: Fig. S1). Pattern 18 is shared by eight eriophyoid mite species, while patterns 1, 4, 10, 19, 23, 24, 26, 27, 33, 34, 44, 47, 50 and 54 are shared by two to four species respectively. The remaining 38 patterns are seen in one species each. To measure the extent of rearranged mt gene orders, we calculated breakpoints for the 54 patterns. The value of breakpoints ranges from 13 (Pattern 23 and Pattern 25) to 25 (Pattern 42) in the Eriophyidae, from 14 (Pattern 20) to 23 (Pattern 10) in the Diptilomiopidae, and from 11 (Pattern 3) to 18 (Pattern 1) in the Phytoptidae (Additional file 1: Table S2).

### Shared derived mt gene clusters support the monophyly of eriophyoid mites (Eriophyoidea) and six lineages within Eriophyoidea

**Fig. 1:**
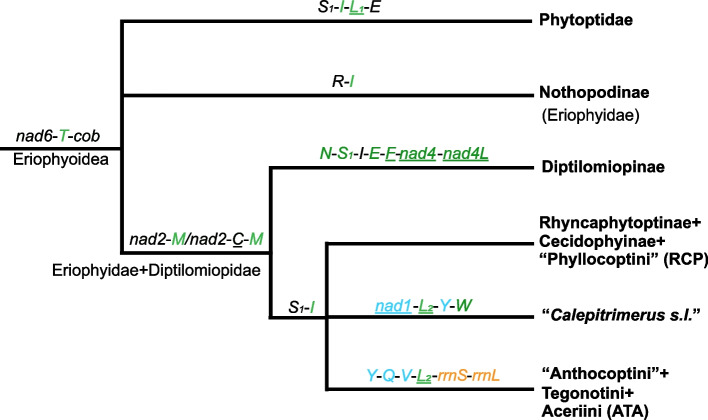
Eriophyoid phylogram inferred by shared derived gene clusters from 153 complete mitochondrial genomes. Genes underlined have opposite transcription orientation to those not underlined. *nad1–6* and *nad4L* for NADH dehydrogenase subunits 1–6 and 4L; *rrnL* and *rrnS* for large and small rRNA subunits; tRNA genes are indicated by the single-letter IUPAC-IUB abbreviations for their corresponding amino acids. Translocated or inverted genes are colour-coded (blue: inversion and translocation; green: translocation; orange: inversion)

Based on shared derived mt gene clusters (Additional file 2: Figs. S1 and S2), we recovered eriophyoid mites (Eriophyoidea) and six lineages within Eriophyoidea as monophyletic (Fig. 1). These shared derived mt gene clusters were analysed in essentially the same way as morphological synapomorphies. We used a parsimony method to group eriophyoid mites based on shared derived mt gene clusters. The gene cluster *nad6-trnT-cob* is derived and shared by all eriophyoid mite species thus supports the monophyly of Eriophyoidea (Fig. 1 and Additional file 2: Fig. S1). The gene cluster *trnS*_*1*_*-trnI-trnL*_*1*_*-trnE* (note: gene underlined has opposite transcription orientation to those not underlined) is derived and shared by all Phytoptidae species thus supports the monophyly of this family. Similarly, *trnR-trnI* is derived and shared by all Nothopodinae species (Fig. 1) and *trnF-trnN-nad5* is derived and shared by all *Cosella* spp. (Additional file 2: Supplementary Fig. S1) thus supporting the monophyly of Nothopodinae and *Cosella*. The gene cluster *nad2-trnM* (or *nad2-trnC-trnM* in four species) is derived and shared by four other lineages, i.e. Diptilomiopinae, Rhyncaphytoptinae + Cecidophyinae + “Phyllocoptini (including 81% represented Phyllocoptini species)” (acronymized as RCP), “Anthocoptini (including 90% represented Anthocoptini species)” + Tegonotini + Aceriini (acronymized as ATA) and “*Calepitrimerus s.l.*”, thus supporting them together as a monophyletic group. Within this group, the shared derived gene cluster *trnN-trnS*_*1*_*-trnI*-*trnE*-*trnF*-*nad4*-*nad4L* (or *trnN-trnE-trnS*_*1*_*-nad4*-*nad4L* in one species and *trnN-trnS*_*1*_*-trnI*-*nad4*-*trnE*-*trnF*-*nad4L* in another species) supports the monophyly of Diptilomiopinae, and the shared derived gene cluster *trnS*_*1*_*-trnI* (or *trnS*_*1*_*-trnE-trnI* in one species and *trnN-trnI* in another species) supports the monophyly of RCP, ATA and “*Calepitrimerus s.l.*” as a group to the exclusion of Diptilomiopinae. The monophyly of ATA is supported by the shared derived gene cluster *trnY-trnQ-trnV*-*trnL*_*2*_*-rrnS-rrnL*, and the monophyly of “*Calepitrimerus s.l.*” is supported by the shared derived gene cluster *nad1-trnL*_*2*_*-trnY-trnW*. For the group ATA, there were further changes to the shared derived gene cluster *trnY-trnQ-trnV*-*trnL*_*2*_*-rrnS-rrnL* in three species: *trnV* and *trnL*_*2*_ swapped their positions in *Tegolophus celtis* (P48; Additional file 2: Fig. S1); *trnS*_*1*_ was inserted between *trnQ* and *trnV* in *Acaphyllisa fagi* (P49; Additional file 2: Fig. S1); both *trnV* and *trnY* translocated in *Epitrimerus c.f. spirae* (P51; Additional file 2: Fig. S1). There is no shared derived gene cluster that supports the monophyly of RCP.

### Phylogeny of the Eriophyoidea inferred from mt genome sequences

**Fig. 2:**
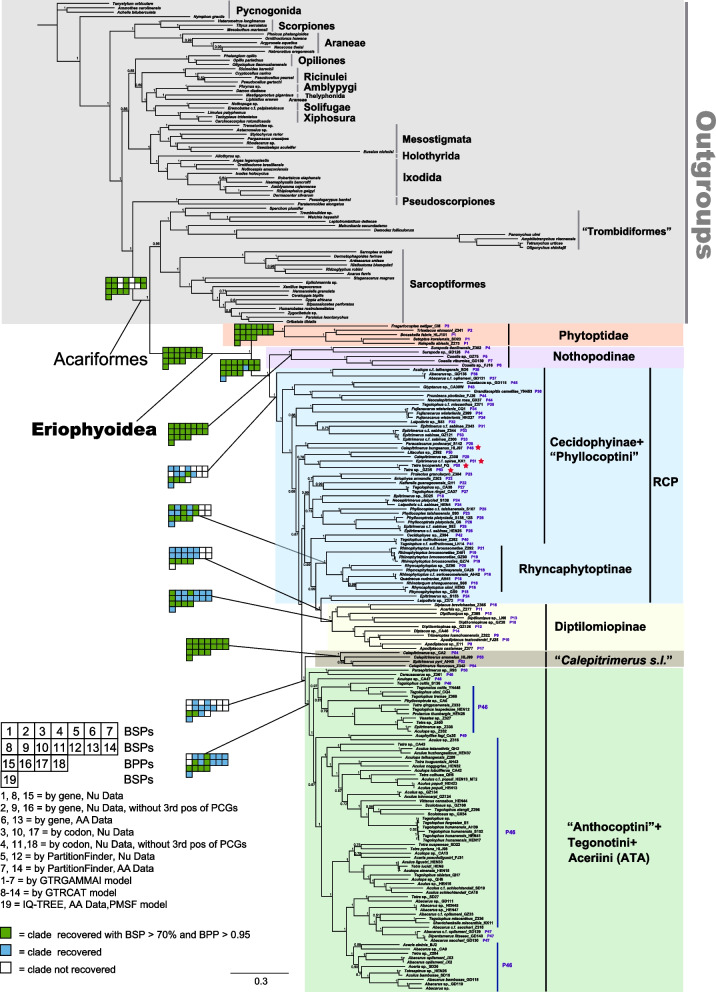
Phylogenetic trees inferred from mitochondrial genome sequences using maximum likelihood and Bayesian methods. The tree topology is largely stable across all analyses; branch lengths presented here follow the Bayesian analysis using nucleotide sequence dataset partitioned by genes. Node numbers indicate Bayesian posterior probabilities (BPP). Maximum likelihood bootstrap proportion (BSP) and BPP values of major clades are shown with colour-coded squares. Green squares indicate clades support with BSP > 70% and BPP > 0.95; blue recovered with moderate or low support; white not recovered. The number of the corresponding mitochondrial genome arrangement pattern is marked after the name of each species (Patterns 1–54) in the Eriophyoidea. Red stars denote genome patterns that were assigned into clade ATA in Fig. 1

Tree topologies inferred from ML and BI analyses were identical, except for a few shallow nodes (Fig. 2, Additional file 2: Figs. S3–S20). The monophyly of superfamily Eriophyoidea was recovered in all trees (Fig. 2, Additional file 2: Figs. S3–S20) with moderate to strong support (BPP > 0.88, BSP = 100). The monophyly of Phytoptidae was recovered with strong support (BPP = 1, BSP = 100). The monophyly of Eriophyidae and Diptilomiopidae was rejected. Two subfamilies, Nothopodinae and Nalepellinae, were recovered as monophyletic groups (Fig. 2) in all trees with strong support (BPP = 1, BSP = 100). The monophyly of Diptilomiopinae was recovered with low to strong support (BPP > 0.98, BSP > 17; Fig. 2). The monophyly of Rhyncaphytoptinae was largely recovered in trees inferred from nucleotide sequence dataset (Fig. 2), while was rejected in trees inferred from amino acid dataset (Additional file 2: Figs. S11, S12, S18, S19). The monophyly of the remaining subfamilies, tribes and all tested genera was rejected (Fig. 2, Additional file 2: Figs. S3–S20). We recovered the monophyly of the group “*Calepitrimerus s.l.*” with strong support (Fig. 2). Our portrayed tree based on shared derived mt gene clusters (Fig. 1) largely mirrors the topologies inferred from mt genome sequences (Fig. 2). However, we found two discrepancies: Rhyncaphytoptinae + Cecidophyinae + “Phyllocoptini” (RCP) (Fig. 1) was recovered as polyphyletic (Fig. 2), and “Anthocoptini” + Tegonotini + Aceriini (ATA) (Fig. 1) was not monophyletic because four species of ATA were mixed with species in the RCP (marked as red stars in Fig. 2). The backbone nodes of RCP are highly unstable (Additional file 2: Fig. S21). Although we largely recovered the monophyly of Rhyncaphytoptinae, we were unable to find shared derived mt gene clusters for this subfamily (Additional file 2: Fig. S1).

### Divergence time of main clades in the Eriophyoidea

**Fig. 3:**
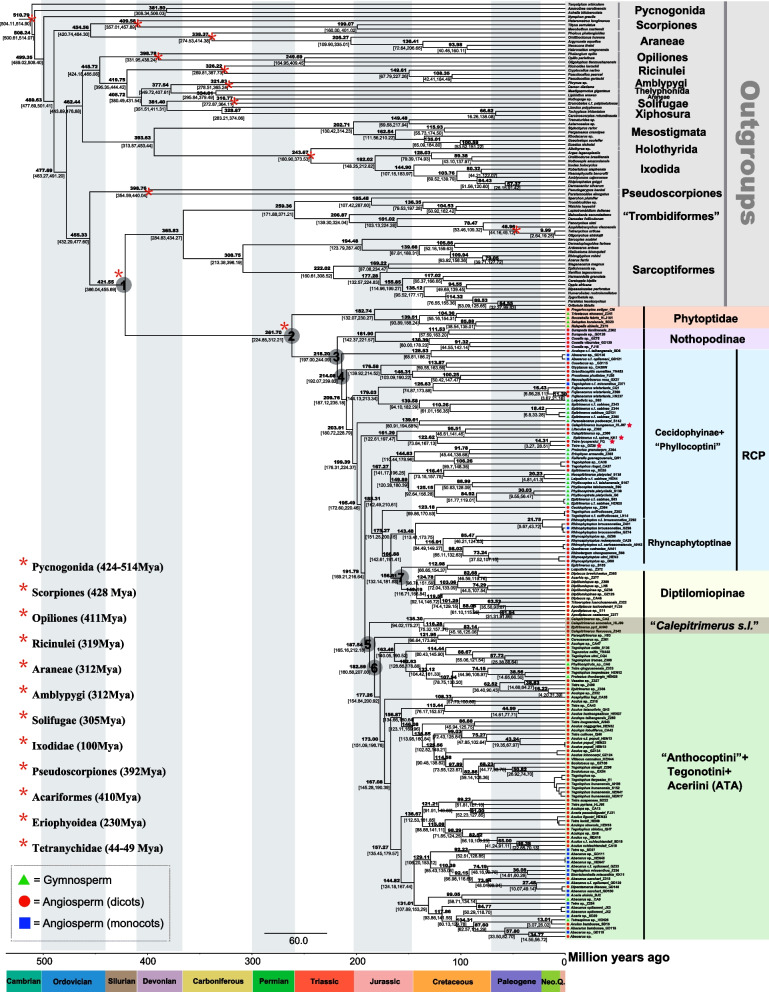
Dated phylogenetic tree of the Eriophyoidea inferred from mitochondrial nucleotide sequence dataset with MCMCTree in PAML. Blue bars at nodes represent 95% highest posterior density (HPD) interval. Numbers above branches represent Bayesian posterior probabilities. Node numbers correspond to the node ages are shown in Table 1. Calibration points are depicted by asterisks

The earliest divergence of Eriophyoidea from the Sarcoptiformes + “Trombidiformes” could be traced back to the middle Silurian (421.55 Ma, 95% HPD 386.04–455.69 Ma; Table 1; Fig. 3). The origin of eriophyoid mites was dated to the late Permian, in line with the divergence of the family Phytoptidae from Eriophyidae + Diptilomiopidae (261.70 Ma, 95% HPD 224.85–312.21 Ma; Fig. 3). The subfamily Nothopodinae diverged from other Eriophyidae + Diptilomiopidae species in the late Triassic (218.20 Ma, 95% HPD 197.00–244.09 Ma; Fig. 3). Eriophyoid mites in the clades RCP, Diptilomiopinae, “*Calepitrimerus s.l.*” and ATA diverged from each other in the Jurassic (Table 1; Fig. 3). After the formation of the main clades of eriophyoid mites, further species explosion events were concentrated in the Cretaceous (Fig. 3), coinciding with the diversification of angiosperms [39].

**Table 1:** Age of the clades (nodes) in Fig. 3

Node	Age Ma (95% HPD)
1	421.55 (386.04–455.69)
2	261.70 (224.85–312.21)
3	218.20 (197.00–244.09)
4	214.08 (192.07–239.82)
5	187.54 (160.58–212.18)
6	182.59 (160.58–207.00)
7	156.81 (132.14–181.63)

Dated phylogenetic trees were constructed using MCMCTree in PAML

*Ma* million years ago, *HPD* highest posterior density

### High correlations between the extent of changed mt gene orders and the rate of nucleotide substitutions in Arachnida

**Fig. 4:**
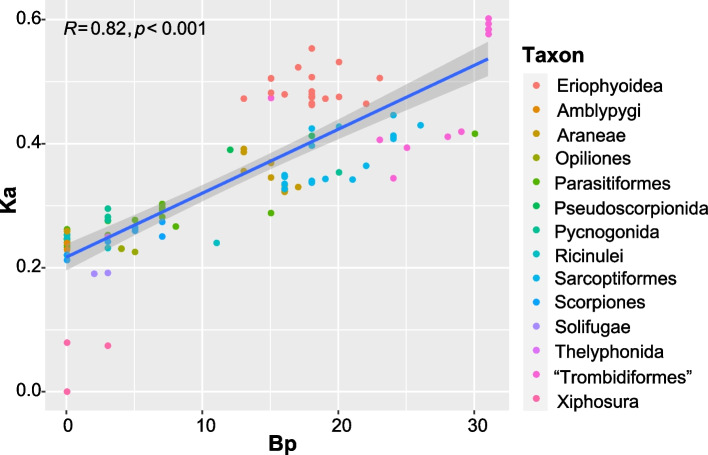
The correlations between the rate of nucleotide substitutions (*Ka*) and breakpoints (Bp) in 111 arachnid species. Arachnid lineages were marked by different colours

Nucleotide sequences analysis of PCGs showed that Eriophyoidea and “Trombidiformes” have the highest nucleotide substitution rates (Additional file 2: Fig. S22a), followed by Araneae, Pseudoscorpines and Sarcoptiformes (*Ka* > 0.3), while the remaining orders (i.e. Pycnogonida, Parasitiformes, Thelyphonida, Scorpiones, Ricinulei, Amblypygi, Opiliones, Solifugae; Additional file 2: Fig. S22a) have relatively low values (*Ka* < 0.3). In Arachnida, highly rearranged mt genomes occurred in the “Trombidiformes,” Sarcoptiformes, Eriophyoidea, Araneae and Pseudoscorpines (breakpoints > 15; Additional file 2: Fig. S22b); the other orders have relatively modest to no rearrangements (breakpoints ranged from 0 to 10; Additional file 2: Fig. S22b). We plotted the correlation between breakpoints (Bp) and the rate of nucleotide substitutions (*Ka*) for 112 arachnid species; there was a significantly positive correlation between them (Pearson R = 0.82, df = 110, *p* < 0.001; Fig. 4).

## Discussion

### Derived mt gene clusters help resolve the higher-level phylogeny of eriophyoid mites

The higher-level phylogeny of eriophyoid mites (Eriophyoidea) has been challenging for over half a century [4, 12] due to homoplasy of morphological characters [11]. In this study, we sequenced and compared the complete mt genomes of 153 eriophyoid mite species, covering all three families, eight subfamilies, 13 tribes and 48 genera. All obtained mt genomes show high rearrangements in gene order relative to the hypothetical ancestral mt gene arrangement of arthropods [38]. We recovered 54 arrangement patterns from 153 mt genomes (Additional file 2: Fig. S1). The shared derived mt gene clusters support the monophyly of eriophyoid mites (Eriophyoidea) as a whole and the monophyly of six clades within Eriophyoidea (Fig. 1). In general, the monophyletic groups and their relationships based on derived mt gene clusters (Fig. 1) were largely supported in the phylogenetic trees inferred from mt genome sequences (Fig. 2), indicating the utility of rearranged mt gene orders in the phylogenetic reconstruction of eriophyoid mites.

Our portrayed tree (Fig. 1) is partly consistent with the morphology-based classification system [4]. Phytoptidae was recovered as monophyletic in the current study (Figs. 2 and 3). The monophyly of Phytoptidae was also supported by a few potential morphological synapomorphies (i.e. setae *vi*, *ve*, *c1*; following Amrine et al. [4]). The current study included only five phytoptid species. It should be noted that Phytoptidae was rendered as polyphyletic in previous molecular studies that included more phytoptid species (nearly 50 species) [17, 40, 41]. The monophyly of the subfamily Nothopodinae was supported previously by morphological characters (i.e. tibiae fused with tarsi, following Amrine et al. [4]), and was supported in the current study by mt genome sequence (Fig. 2) and shared derived gene cluster analyses (Fig. 1). The monophyly of Diptilomiopinae was also supported previously by morphological characters (i.e. gnathosoma large, chelicerae abruptly curved and bent down near base, and empodium divided; following Amrine et al. [4]), and by shared derived mt gene clusters (Fig. 1) and mt genome sequence analyses in the current study (Fig. 2). No morphological synapomorphies were obtained for the RCP, ATA and “*Calepitrimerus s.l.*”. Although we demonstrated the use of derived mt gene clusters in resolving the higher-level phylogeny of eriophyoid mites, the phylogeny inferred from mt genome sequences should be used with caution. For example, RCP was rendered as polyphyletic by mt genome sequence analysis (Fig. 2) as well as some unstable internal nodes (Additional file 2: Fig. S21). ATA was not monophyletic because four species of ATA were mixed with species in the RCP (marked as red stars; Fig. 2). Furthermore, mt genome sequences showed limited ability in resolving the order-level phylogeny of arachnids [30]. Future resolution of the phylogeny of eriophyoid mites requires more data, such as whole genome or whole transcriptome sequences (e.g. ultra-conserved elements; [42]), and other discrete characters (e.g. karyotype and homologous genes) inferred by synteny approach [43–45].

In line with previous molecular studies [11, 17, 40] as well as our mt gene order evidence, we argue for a revision of the classification system of Eriophyoidea to reflect their phylogeny. Furthermore, the monotypic or species poor subfamilies (Prothricinae, Novophytoptinae, Aberoptinae, Ashieldophyinae) and tribes (Pentasetacini, Mackiellini, Colopodacini, Diphytoptini, Adventacarini), not included in the current study, should be determined in future studies to decipher their phylogenetic positions.

### Potential factors for highly rearranged mt genomes in Arachnida

The exact factors that drive the rearrangement of mt genes remain unclear. Our results show strong correlations between the extent of changed gene orders (breaking points) and the rates of nucleotide substitutions (host evolutionary rates; *p* < 0.001; Fig. 4). High values of nucleotide substitution are found in lineages of Trombidiformes, Sarcoptiformes, Eriophyoidea, Araneae and Pseudoscorpines (Additional file 2: Fig. S22a). These lineages were inferred as having high evolution rates (long-branches) in phylogenomics studies [46, 47]. Furthermore, these lineages, by consisting of relatively large number of species in the Arachnida, may indirectly reflect their high speciation rates [30]. We therefore suggest that high evolutionary rates of arachnid species might trigger their highly diversified mt genome patterns during long-term coevolution. Nevertheless, many other factors for inducing mt gene rearrangement should be determined in the further studies, such as mt genome recombination [48] or the interactions of mitochondria and nuclear genome (haplodiploid nuclear genetics in eriophyoid mites [49]).

### Evolutionary trajectory of eriophyoid mites with their plant hosts

Eriophyoid mites were thought to have an ancient origin, dated back to the Triassic [50]. Our dated results showed that the origin of crown eriophyoid mites was dated to the late Permian (261.70 Ma, Fig. 3) and was largely consistent with previous suggestions [17, 36, 40]. Our inferred major clades diverged in the Jurassic and Cretaceous periods (Fig. 3), thus are in line with the emergence/divergence of angiosperms [51, 52]. Because eriophyoid mites are strictly phytophagous and have high host specificity (i.e. 80% species are monophagous) [53, 54], we therefore expect that host plant preference (i.e. phylogenetic niche conservatism) may influence the diversity of eriophyoid mites in the light of their long-term coevolution. Our phylogenetic trees provide evidence that eriophyoid mites are largely grouped by host plants, i.e. gymnosperms, monocots and dicots (Fig. 3). These findings are consistent with a previous study [17]. Eriophyoid mites were suggested to have an ancient diet on gymnosperms [50], then expanded to angiosperms by multiple host shifts [17]. Host plant expansion might trigger the species diversity of eriophyoid mites during long-term coevolution. Collectively, we herein speculate three key periods reflecting the evolutionary trajectory of eriophyoid mites with host plants (Fig. 3): (1) the origin of eriophyoid mites in late Permian period (the mass end-Permian extinction, gymnosperms dominance); (2) major clade formation in the Jurassic period; and (3) explosive speciation of eriophyoid mite species with the flourishing of angiosperms in the Cretaceous to the present (angiosperms dominance).

## Conclusions

In the current study, we showed multiple molecular synapomorphies (i.e. shared derived mt gene clusters) at different levels (i.e. family, subfamily or tribe levels) from the complete mt genomes of 153 eriophyoid mite species. These synapomorphies served as keys to resolve the higher-level phylogeny of Eriophyoidea. The extent of changed mt gene orders tightly links to the evolutionary rates of arachnid lineages. However, other factors should be determined in future studies, such as mt genome recombination [48] and haplodiploid nuclear genetics [49]. Our results highlight the utility of mt gene rearrangement for resolving the higher-level phylogeny of eriophyoid mites. In addition to mt genomes, more data (e.g. nuclear genomes, karyotype and homologous genes) should be explored in future phylogenetic analysis of the Eriophyoidea. We herein suggest a revision of the classification system among the Eriophyoidea.

## Methods

### Taxon sampling

We compiled a dataset including 153 eriophyoid mite terminals (139 putative species, Additional file 1: Table S1) and 74 outgroup species (Additional file 1: Table S3 [27, 29, 30, 55–91]). The complete mt genomes of 148 eriophyoid mites were newly sequenced, while the remaining mt genomes of five eriophyoid mite species and 74 outgroups were retrieved from GenBank (Additional file 1: Table S3). The nomenclature for taxa and classification of Eriophyoidea follows Amrine et al. [4]. Most of the missing subfamilies and tribes are species poor. Outgroups include 27 Acariformes species, two Amblypygi species, six Araneae species, three Opiliones species, 17 Parasitiformes species, two Pseudoscorpiones species, four Pycnogonida species, four Ricinulei species, three Scorpiones species, two Solifugae species, one Thelyphonida species and three Xiphosura species. Since the monophyly of Acariformes was consistently found in previous studies [92–96], we therefore used non-acariform species as remote outgroups. Sea spiders were used to root the tree. All our samples were preserved in 96% ethanol at − 20 °C until DNA extraction. Samples of each species were also slide-mounted as vouchers, using modified Berlese medium for morphological checking with a Zeiss A2 microscope. All specimens and vouchers were deposited in the Arthropod Collection, Department of Entomology, Nanjing Agricultural University, China.

### Mitochondrial genome sequencing of eriophyoid mites

Since eriophyoid mite species are especially tiny (~ 200 um in length), the low quantity of genomic DNA extracted from individual mites hampered direct Illumina sequencing. We therefore adopted a new approach using multiple displacement amplifications.

1. The genomic DNA of one mite individual was extracted for each sample, using a DNeasy Blood and Tissue Kit (Qiagen, Germany), followed by a previously reported protocol [97]. A 658-bp fragment of the *cox1* gene for each species was amplified by PCR with the primer pairs LCO1490–HCO2198 [98]. The PCR amplicons were purified and sequenced directly using Sanger method at General Biological Company (Nanjing, China).
2. The entire mitochondrial genomes of each sample with mixed species (i.e. five species pooled, 20 individuals per species) were amplified using REPLI-g® Mitochondrial DNA Kit (Qiagen, Germany). We modified the amplification process by using specific primers for eriophyoid mites (Additional file 1: Table S4) according to the User-Developed Protocol. These specific primers were derived from previously sequenced four mt genomes of eriophyoid mites (i.e. *Leipothrix juniperensis*, *Epitrimerus sabinae*, *Phyllocoptes taishanensis* and *Rhinotergum shaoguanense*) [35, 36, 99]. Amplicons were purified by VAHTS Universal Plus DNA Library Prep Kit (Vazyme, China). A 350-bp paired-end library was constructed and sequenced by Illumina Hiseq 2000 platform at Personalbio Company (Shanghai, China). A total of 3 Gb of data was obtained for each library.
3. Mitochondrial genomes were assembled by GetOrganelle 1.7.1 with default parameters [100], using the sequence of the *cox1* fragment as a reference and were annotated following previous studies [35, 36].

### Phylogenetic analyses

The nucleotide sequences and amino acid sequences of protein-coding genes (PCGs) were aligned individually with TranslatorX web server (http://translatorx.co.uk/) [101] using MAFFT version 7 [102] to compute the alignments based on translated protein sequences. Large gaps and ambiguous sites were deleted with Gblocks v0.91 [103] using parameters for a less stringent selection such as “Maximum number of contiguous non-conserved positions = 5”, “Minimum length of a block = 2” and “Allowed gap positions = all”. Two rRNA genes (*rrnS* & *rrnL*) were aligned using MUSCLE implemented in MEGA X [104]; ambiguous sites and large gaps were also deleted with Gblocks v0.91. In total, 11,121 bp of nucleotide sites and 3544 bp of amino acid sites were removed. Nucleotide sequence alignments of 15 genes (13 PCGs and two rRNAs) and amino acid sequence alignments of 13 PCGs genes were concatenated with SeqKit [105]. We compiled three dataset matrices: (1) nucleotide sequences (10,071 bp), (2) nucleotide sequences excluding the third codon positions of PCGs (7,153 bp) and (3) amino acid sequences (2268 amino acids). The nucleotide sequence matrix was partitioned by genes (13 PCGs and two rRNAs) or by codons (three for 13 PCGs) and two rRNA genes, while the amino acid matrix was partitioned by genes (13 partitions, Additional file 1: Table S5). All dataset matrices were partitioned by PartitionFinder2 [106], using *unlinked* branch lengths, the *greedy search* algorithm, and *MrBayes* or *Raxml* model (Additional file 1: Table S5). The substitution model GTR + I + G was chosen by PartitionFinder as the best for three of five partitions, and HKY + G and HKY + I + G models for the other two partitions.

Phylogenetic analyses were conducted using maximum likelihood (ML) and Bayesian inference (BI) methods. ML analyses were performed with RaxML-HPC2 [107] and IQ-TREE 2.2.2.6 [108]. In RaxML-HPC2, all datasets were run twice using the GTRGAMMAI model and GTRCAT model separately in the RaxML-HPC2 on XSEDE [107] through the CIPRES Science Gateway V3.3 [109]. Clade support was generated using a nonparametric bootstrap with 1000 replicates. In IQ-TREE 2.2.2.6, the dataset of amino acid sequences was run with the posterior mean site frequency (PMSF) model, using the LG + C20 + F + Γ implementation, to avoid long-branch attraction artifacts [110]. A guide tree was inferred using the LG4X mixture model in IQ-TREE 2.2.2.6 [108]. The bootstrap values (BSP) ≥ 70% were considered as strong support for specific phylogenetic relationships [111]. BI analyses were performed with MrBayes 3.2.6 [112]. In MrBayes 3.2.6, all data sets were run with mixed models (Additional file 1: Table S5). The combined dataset was run for 20 million generations, with trees sampled every 1000 generations. The average standard deviation gradually drops below 0.01 in most Bayesian trees after 0.8 million generations. The Bayesian posterior probabilities (BPP) ≥ 95% were considered as strong support for specific phylogenetic relationships [113]. All tree files were edited by FigTree v1.4.3 [114].

### Divergence time estimation

We estimated the divergence time of eriophyoid mite species using MCMCTree in PAML v.4.9 [115] by dataset of nucleotide sequences (10,071 bp) with the approximate-likelihood method [116]. The input tree topology was fixed as the Fig. 2 inferred from BI. MCMCTree used a birth–death-sampling speciation prior and were run 5 × 10^5^ generations, sampling one tree by every ten generations after discarding 20% generations as burn-in. We selected twelve fossil calibrations, reflecting their oldest known fossils. All calibrations were used as soft minimum bounds (using uniform distributions with informative maximum ages). These ages were used as the minimum soft boundaries of the corresponding lineage nodes, and this way of calibration avoids problems caused by the uncertainty of fossils [115]. The first constraint was set for the crown Pycnogonida with a minimum age of 424 Ma and a maximum age of 514 Ma [30, 117, 118]. The second constraint was set for the crown Scorpiones with a minimum age of 428 Ma and a maximum age of 514 Ma [30, 117, 119]. The crown of Opiliones was set as the third constraint to a minimum age of 411 Ma and a maximum age of 514 Ma [30, 117, 120, 121]. The fourth was used to the crown Ricinulei with a minimum age of 319 Ma and a maximum age of 514 Ma [30, 117, 122]. The fifth was used to the crown Araneae with a minimum age of 312 Ma and a maximum age of 514 Ma [30, 117]. The crown of Amblypygi was set as the sixth with a minimum age of 312 Ma and a maximum age of 514 Ma [30, 117]. The seventh fossil calibration was set for the crown Solifugae with a minimum age of 305 Ma and a maximum age of 514 Ma [30, 117, 123]. The crown of Ixodidae was set as the eighth constraint to a minimum age of 100 Ma and a maximum age of 514 Ma [30, 117]. Pseudoscorpiones was set as the ninth with a minimum age of 392 Ma and a maximum age of 514 Ma [30, 117]. The tenth was used to the crown Acariformes with a minimum age of 410 Ma and a maximum age of 514 Ma [30, 117, 124]. The eleventh fossil calibration was set for the crown Eriophyoidea with the minimum age of 230 Ma and a maximum age of 410 Ma [30, 50, 125]. Tetranychidae in Trombidiformes was set as the last with a minimum age of 44 Ma and a maximum age of 49 Ma [30, 117]. Tracer v1.7.2 [126] was used to confirm the stationary distribution of acceptable mixing of the Markov Chain Monte Carlo (MCMC) steps and ensure that each parameter had been appropriately sampled (ESS > 200). The posterior probability was considered to be a measure of node support. The consensus tree was edited by FigTree v1.4.3. [114].

### Correlations between the extent of rearranged mt gene orders and the rates of nucleotide substitutions in the Arachnida

To test the correlations of the extent of changed mt gene orders with the substitution rates of PCGs, we assembled an additional dataset including 110 species from 11 arachnid lineages (i.e. Amblypygi, Araneae, Eriophyoidea, Opiliones, Parasitiformes, Pseudoscorpines, Pycnogonida, Ricinulei, Sarcoptiformes, Scorpiones, Solifugae, Trombidiformes, Thelyphonida and Xiphosura; Additional file 1: Table S6). Horse-shoe crabs of the genus *Limulus* were regarded as “living fossils” of the Chelicerata, reflecting potential ancestral mt gene arrangement [38]. We therefore used the mt genome of *Limulus polyphemus* as a conservative species to calculate the corresponding breakpoints, *Ka* (number of nonsynonymous substitutions per nonsynonymous site) and *Ks* (number of synonymous substitutions per synonymous site) to the remaining species.

Breakpoints were calculated with CREx [127] web server (http://pacosy.informatik.uni-leipzig.de/crex) as a measure of the extent of mt gene rearrangement. *Ka* and *Ks* were calculated to measure the rates of sequence substitution for PCGs using DnaSP 6.0 [128]. Because *Ks* of all species reached a saturation, *Ka* was used as an alternative proxy (Additional file 1: Table S6). The correlations between the extent of gene rearrangements (breakpoints) and the rates of nucleotide substitutions (*Ka*) were measured using R packages ‘ggpubr’ and were plotted in ‘ggplot2’ [129, 130]. 

### Supplementary Information

**Additional file 1:** **Table S1.** Eriophyoid mite species included in this study [35,36,37].**Table S2.** Breakpoints of eriophyoid mitochondrial genomes with different gene arrangement patterns. **Table S3.** Outgroups used in phylogenetic analysis based on mitochondrial genome sequences [27, 29, 30, 55, 56, 57, 58, 59, 60, 61, 62, 63, 64, 65, 66, 67, 68, 69, 70, 71, 72, 73, 74, 75, 76, 77, 78, 79, 80, 81, 82, 83, 84, 85, 86, 87, 88, 89, 90, 91]. **Table S4.** Sequence of primers used in this study. **Table S5.** Datasets used in phylogenetic analysis through different partitions. **Table S6.** Arachnida species included in this study in correlation analysis between rate of gene rearrangement and rate of nucleotide substitution.

**Additional file 2:** **Fig. S1.** The 54 mitochondrial gene arrangement patterns (Pattern 1–Pattern 54) from 153 eriophyoid mite species in this study. Genes underlined have opposite transcription orientation to those not underlined. Translocated or inverted genes are colour‐coded (blue: inversion and translocation; green: translocation; orange: inversion). Abbreviations of protein‐coding genes are *atp6 *and* atp8* for ATP synthase subunits 6 and 8; *cox1–3* for cytochrome oxidase subunits 1–3; *cob *for cytochrome b; *nad1–6* and *nad4L* for NADH dehydrogenase subunits 1–6 and 4L; *rrnL* and *rrnS* for large and small rRNA subunits; tRNA genes are indicated by the single‐letter IUPAC‐IUB abbreviations for their corresponding amino acids. The shared rearranged mt gene clusters of each sample were denoted by underneath lines in different colors as the same as Fig. 1.** Fig. S2.** Mitochondrial gene arrangements of representative samples in the Eriophyoidea. Two samples were selected as representatives of each clade. Shared rearranged mt gene clusters of each sample were denoted by underneath lines in different colors. Underlined genes are encoded in the N‐strand. Translocated or inverted genes are colour-coded (blue: inversion and translocation; green: translocation; orange: inversion). Abbreviations of protein‐coding genes are *atp6* and atp8 for ATP synthase subunits 6 and 8; *cox1–3* for cytochrome oxidase subunits 1–3; cob for cytochrome b; *nad1–6* and *nad4L* for NADH dehydrogenase subunits 1–6 and 4L; *rrnL *and *rrnS *for large and small rRNA subunits; tRNA genes are indicated by the single‐letter IUPAC‐IUB abbreviations for their corresponding amino acids.**Fig. S3.** The phylogenetic tree inferred from mitochondrial genome nucleotide sequences using maximum likelihood method with partition by gene and GTRGAMMAI model.** Fig. S4.** The phylogenetic tree inferred from mitochondrial genome nucleotide sequences (without the 3rd codon positions of PCGs) using maximum likelihood method with partition by gene and GTRGAMMAI model.** Fig. S5.** The phylogenetic tree inferred from mitochondrial genome nucleotide sequences using maximum likelihood method with partition by codon and GTRGAMMAI model.** Fig. S6.** The phylogenetic tree inferred from mitochondrial genome nucleotide sequences (without the 3rd codon positions of PCGs) using maximum likelihood method with partition by codon and GTRGAMMAI model.** Fig. S7.** The phylogenetic tree inferred from mitochondrial genome nucleotide sequences using maximum likelihood method with partition by PartitionFinder and GTRGAMMAI model.** Fig. S8.** The phylogenetic tree inferred from mitochondrial genome amino acid sequences using maximum likelihood method with partition by gene and GAMMA model.** Fig. S9.** The phylogenetic tree inferred from mitochondrial genome amino acid sequences using maximum likelihood method with partition by PartitionFinder and GAMMA model.** Fig. S10.** The phylogenetic tree inferred from mitochondrial genome nucleotide sequences using maximum likelihood method with partition by gene and GTRCAT model.** Fig. S11.** The phylogenetic tree inferred from mitochondrial genome nucleotide sequences (without the 3rd codon positions of PCGs) using maximum likelihood method with partition by gene and GTRCAT model.** Fig. S12.** The phylogenetic tree inferred from mitochondrial genome nucleotide sequences using maximum likelihood method with partition by codon and GTRCAT model.** Fig. S13.** The phylogenetic tree inferred from mitochondrial genome nucleotide sequences (without 3rd codon positions of PCGs) using maximum likelihood method with partition by codon and GTRCAT model.** Fig. S14.** The phylogenetic tree inferred from mitochondrial genome nucleotide sequences using maximum likelihood method with partition by PartitionFinder and GTRCAT model.** Fig. S15.** The phylogenetic tree inferred from mitochondrial genome amino acid sequences using maximum likelihood method with partition by gene and GTRCAT model.** Fig. S16.** The phylogenetic tree inferred from mitochondrial genome amino acid sequences using maximum likelihood method with partition by PartitionFinder and GTRCAT model.** Fig. S17.** The phylogenetic tree inferred from mitochondrial genome nucleotide sequences (without 3rd codon positions of PCGs) using Bayesian method and partition by gene.** Fig. S18.** The phylogenetic tree inferred from mitochondrial genome nucleotide sequences using Bayesian method and partition by codon.** Fig. S19.** The phylogenetic tree inferred from mitochondrial genome nucleotide sequences (without 3rd codon positions of PCGs) using Bayesian method and partition by codon. **Fig. S20. **The phylogenetic tree inferred from mitochondrial amino acid sequences using PMSF method in IQ-TREE with LG + C20+F+ G” model. **Fig. S21. **The relevant Navajo (sensitivity) plots to the 12 internal nodes of the RCP grade in Fig. 2. **Fig. S22. **(a) Box plots of*Ka* (nonsynonymous substitutions per nonsynonymous site) in different arachnid lineages, marked by different colours. (b) Box plots of Bp (breakpoints) in different arachnid lineages, marked by different colours.

**Additional file 3.** Original untrimmed alignments by Gblocks v0.91.

## Data Availability

Datasets, alignments and phylogenetic trees available from Dryad Digital Repository: 10.5061/dryad.612jm647k [131]. GenBank Accessions numbers are given in Additional file 1: Table S1. Original untrimmed alignments by Gblocks v0.91 are provided in Additional file 3.

## Abbreviations

Mt: Mitochondrial
PCGs: Protein-coding genes
rRNAs: Ribosomal RNA genes
tRNAs: Transfer RNA genes
rrnS: 12S ribosomal RNA
rrnL: 16S ribosomal RNA
nad2: NADH dehydrogenase subunits 2
nad4: NADH dehydrogenase subunits 4
nad4L: NADH dehydrogenase subunits 4L
nad5: NADH dehydrogenase subunits 5
nad6: NADH dehydrogenase subunits 6
cox1: Cytochrome oxidase subunits 1
cob: Cytochrome b
ML: Maximum likelihood methods
BI: Bayesian methods
BPP: Bayesian posterior probabilities
BSP: Maximum likelihood bootstrap proportion
HPD: Highest posterior density
Ka: Number of nonsynonymous substitutions per nonsynonymous site
Ks: Number of synonymous substitutions per synonymous site
Bp: Breakpoints
K/Pg: The Cretaceous–Paleogene extinction event
