# Supplementary material for: Phylogenomics resolves the higher-level phylogeny of herbivorous eriophyoid mites (Acariformes: Eriophyoidea)
Source: BMC Biol. 2024 Mar 22;22:70. doi: 10.1186/s12915-024-01870-9 (PMC10960459; doi:10.1186/s12915-024-01870-9)
Supplement: Supplementary file 1 — Additional file 1: Table S1. Eriophyoid mite species included in this study [35,36,37].Table S2. Breakpoints of eriophyoid mitochondrial genomes with different gene arrangement patterns. Table S3. Outgroups used in phylogenetic analysis based on mitochondrial genome sequences [27, 29, 30, 55, 56, 57, 58, 59, 60, 61, 62, 63, 64, 65, 66, 67, 68, 69, 70, 71, 72, 73, 74, 75, 76, 77, 78, 79, 80, 81, 82, 83, 84, 85, 86, 87, 88, 89, 90, 91]. Table S4. Sequence of primers used in this study. Table S5. Datasets used in phylogenetic analysis through different partitions. Table S6. Arachnida species included in this study in correlation analysis between rate of gene rearrangement and rate of nucleotide substitution. [file 12915_2024_1870_MOESM1_ESM.docx]

Additional file 1: Table S1. Eriophyoid mite species included in this study

| **Family** | **Subfamily** | **Tribe** | **Species** | **Specimen number** | **Clade** | **Pattern** | **Host plants** | **Size (bp)** | **GenBank**  **number** |
| --- | --- | --- | --- | --- | --- | --- | --- | --- | --- |
| Phytoptidae | Nalepellinae | Nalepellini | *Nalepella abiesis* | Z275 | Phytoptidae | Pattern 1 | *Torreya grandis* Fortune ex Lindl. (Taxaceae) | 14236 | OQ615704 |
|  |  |  | *Setoptus koraiensis* | SD23 | Phytoptidae | Pattern 1 | *Pinus* sp. (Pinaceae) | 14746 | OQ615699 |
|  |  | Trisetacini | *Boczekella fabris* | HLJ101 | Phytoptidae | Pattern 1 | *Larix gmelinii* (Rupr.) Kuzen. (Pinaceae) | 14673 | OQ615674 |
|  |  |  | *Trisetacus ehmanni* | Z341 | Phytoptidae | Pattern 2 | *Pinus* sp. (Pinaceae) | 14944 | OQ615723 |
|  | Sierraphytoptinae | Sierraphytoptini | *Fragariocoptes setiger* | Klimov, et al. [37] | Phytoptidae | Pattern 3 | *Fragaria viridis* (Rosaceae) | 14109 | CM034476 |
| Eriophyidae | Cecidophyinae | Colomerini | *Cosetacus* sp. | GD115 | RCP | Pattern 45 | *Mucuna macrocarpa* Wall. (Fabaceae) | 13997 | OQ615623 |
|  |  | Cecidophyini | *Glyptacus* sp*.* | CA39W | RCP | Pattern 43 | *Quercus robur* L. (Fagaceae) | 14031 | OQ615607 |
|  |  |  | *Cecidophyes* sp. | Z394 | RCP | Pattern 42 | *Castanopsis eyrei* (Champ. ex Benth.) Hutch. (Fagaceae) | 13666 | OQ615736 |
|  | Eriophyinae | Aceriini | *Aceria pseudoligustri* | FJ31 | ATA | Pattern 46 | *Ligustrum lucidum* W.T.Aiton (Oleaceae) | 13571 | OQ615619 |
|  |  |  | *Aceria aloinis* | BJ2 | ATA | Pattern 46 | *Aloe vera* (L.) Burm.f. (Asphodelaceae) | 13466 | OQ615597 |
|  |  |  | *Aceria* sp. | SD20 | ATA | Pattern 46 | *Setaria viridis* (L.) P.Beauv. (Poaceae) | 13678 | OQ615697 |
|  |  | Eriophyini | *Eriophyes armandis* | Z303 | RCP | Pattern 23 | *Pinus* sp. (Pinaceae) | 13446 | OQ615712 |
|  | Nothopodinae | Nothopodini | *Cosella viburniae* | GD139 | Nothopodinae | Pattern 7 | *Viburnum odoratissimum* Ker Gawl. (Adoxaceae) | 13753 | OQ615631 |
|  |  |  | *Cosella* sp. | FJ16 | Nothopodinae | Pattern 6 | *Viburnum chinshanense* Graebn. (Adoxaceae) | 13623 | OQ615616 |
|  |  |  | *Cosella* sp. | GZ75 | Nothopodinae | Pattern 5 | *Viburnum* sp. (Adoxaceae) | 13701 | OQ615640 |
|  |  |  | *Surapoda tianlinensis* | Z302 | Nothopodinae | Pattern 4 | *Cinnamomum camphora* (L.) J.Presl (Lauraceae) | 13626 | OQ615711 |
|  |  |  | *Surapoda* sp. | GD126 | Nothopodinae | Pattern 4 | *Litsea* *pungens* Hemsl. (Lauraceae) | 13749 | OQ615627 |
|  | Phyllocoptinae | Acaricalini | *Dipentamerus litseae* | GD140 | ATA | Pattern 47 | *Litsea glutinosa* (Lour.) C.B.Rob. (Lauraceae) | 13507 | OQ615632 |
|  |  |  | *Acaphyllisa fagi* | CA35 | ATA | Pattern 49 | *Fagus sylvatica* L. (Fagaceae) | 13584 | OQ615604 |
|  |  |  | *Litaculus* sp. | Z392 | RCP | Pattern 30 | *Machilus thunbergii* Siebold & Zucc. (Lauraceae) | 13667 | OQ615735 |
|  |  | Calacarini | *Paracalacarus podocarpi* | S142 | RCP | Pattern 28 | *Podocarpus macrophyllus* (Thunb.) Sweet (Podocarpaceae) | 13560 | OQ615690 |
|  |  | Anthocoptini | *Abacarus bambusae* | GD119 | ATA | Pattern 46 | Bambusoideae (Poaceae) | 13471 | OQ615625 |
|  |  |  | *Abacarus bambusae* | GD118 | ATA | Pattern 46 | *Mucuna birdwoodiana* Tutcher (Fabaceae) | 13529 | OQ615624 |
|  |  |  | *Abacarus oplismeni* | JX2 | ATA | Pattern 46 | Bambusoideae (Poaceae) | 13475 | OQ615676 |
|  |  |  | *Abacarus oplismeni* | JX3 | ATA | Pattern 46 | Bambusoideae (Poaceae) | 13482 | OQ615677 |
|  |  |  | *Abacarus* c.f. *oplismeni* | GZ33 | ATA | Pattern 46 | Bambusoideae (Poaceae) | 13470 | OQ615636 |
|  |  |  | *Abacarus* c.f. *oplismeni* | GD129 | ATA | Pattern 47 | *Oplismenus undulatifolius* (Ard.) Roem. & Schult. (Poaceae) | 13505 | OQ615628 |
|  |  |  | *Abacarus* c.f. *oplismeni* | GD121 | RCP | Pattern 37 | *Oplismenus undulatifolius* (Ard.) Roem. & Schult. (Poaceae) | 13669 | OQ615626 |
|  |  |  | *Abacarus sacchari* | GD130 | ATA | Pattern 47 | *Setaria palmifolia* (J.Koenig) Stapf (Poaceae) | 13331 | OQ615629 |
|  |  |  | *Abacarus* c.f. *sacchari* | Z318 | ATA | Pattern 46 | *Saccharum arundinaceum* Retz. (Poaceae) | 13457 | OQ615716 |
|  |  |  | *Abacarus* sp. | GD111 | ATA | Pattern 46 | *Oplismenus undulatifolius* (Ard.) Roem. & Schult. (Poaceae) | 13486 | OQ615622 |
|  |  |  | *Abacarus* sp. | CA9 | ATA | Pattern 46 | *Thuja occidentalis* L. (Cupressaceae) | 13709 | OQ615600 |
|  |  |  | *Abacarus* sp. | HK3 | ATA | Pattern 46 | *Vitex negundo* L. (Lamiaceae) | 13554 | OQ615669 |
|  |  |  | *Abacarus* sp*.* | HEN47 | ATA | Pattern 46 | *Pennisetum centrasiaticum* Tzvelev (Poaceae) | 13470 | OQ615668 |
|  |  |  | *Abacarus* sp. | HEN45 | ATA | Pattern 46 | *Imperata cylindrica* (L.) Raeusch. (Poaceae) | 13470 | OQ615667 |
|  |  |  | *Abacarus* sp. | GD136 | RCP | Pattern 38 | *Digitaria sanguinalis* (L.) Scop. (Poaceae) | 13664 | OQ615630 |
|  |  |  | *Aculops lobuliferus* | CA42 | ATA | Pattern 46 | *Populus tremuloides* Michx. (Salicaceae) | 13589 | OQ615608 |
|  |  |  | *Aculops sinensis* | HEN18 | ATA | Pattern 46 | *Ligustrum × vicaryi* Rehder (Oleaceae) | 13476 | OQ615657 |
|  |  |  | *Aculops taihangensis* | Z289 | ATA | Pattern 46 | *Ailanthus altissima* (Mill.) Swingle (Simaroubaceae) | 13536 | OQ615707 |
|  |  |  | *Aculops* c.f. *taihangensis* | SD6 | RCP | Pattern 39 | *Ailanthus* sp. (Simaroubaceae) | 13538 | OQ615694 |
|  |  |  | *Aculops* sp. | QH8 | ATA | Pattern 46 | *Ligustrum* sp. (Oleaceae) | 13458 | OQ615686 |
|  |  |  | *Aculops* sp. | CA47 | ATA | Pattern 46 | *Acer platanoides* L. (Sapindaceae) | 13508 | OQ615611 |
|  |  |  | *Aculops* sp. | Z332 | ATA | Pattern 46 | *Kadsura heteroclita* (Roxb.) Craib (Schisandraceae) | 13428 | OQ615719 |
|  |  |  | *Aculops* sp. | CA13 | ATA | Pattern 46 | *Acer negundo* L. (Sapindaceae) | 13850 | OQ615601 |
|  |  |  | *Aculus bambusae* | SD15 | ATA | Pattern 46 | *Carallia brachiata* (Lour.) Merr. (Rhizophoraceae) | 13432 | OQ615695 |
|  |  |  | *Aculus coggygriae* | HEN32 | ATA | Pattern 46 | *Cotinus coggygria* Scop. (Anacardiaceae) | 13497 | OQ615662 |
|  |  |  | *Aculus huzhongsalixus* | HEN37 | ATA | Pattern 46 | *Salix alba* L. (Salicaceae) | 13954 | OQ615664 |
|  |  |  | *Aculus ichnocarpi* | GZ124 | ATA | Pattern 46 | *Hypericum monogynum* L. (Hypericaceae) | 13438 | OQ615645 |
|  |  |  | *Aculus ligustri* | HEN33 | ATA | Pattern 46 | *Ligustrum lucidum* W.T.Aiton (Oleaceae) | 13475 | OQ615663 |
|  |  |  | *Aculus populi* | HEN13 | ATA | Pattern 46 | *Populus* sp. (Salicaceae) | 13598 | OQ615653 |
|  |  |  | *Aculus populi* | HEN23 | ATA | Pattern 46 | *Populus przewalskii* Maxim. (Salicaceae) | 13488 | OQ615658 |
|  |  |  | *Aculus* c.f. *populi* | HEN13_2 | ATA | Pattern 46 | *Populus przewalskii* Maxim. (Salicaceae) | 13599 | OQ615654 |
|  |  |  | *Aculus schlechtendali* | CA18 | ATA | Pattern 46 | *Malus pumila* Mill. (Rosaceae) | 13472 | OQ615602 |
|  |  |  | *Aculus* c.f. *schlechtendali* | SD19 | ATA | Pattern 46 | *Malus baccata* (L.) Borkh. (Rosaceae) | 13459 | OQ615696 |
|  |  |  | *Aculus tetanothrix* | QH2 | ATA | Pattern 46 | *Salix babylonica* L. (Salicaceae) | 14151 | OQ615683 |
|  |  |  | *Aculus* sp. | HEN16 | ATA | Pattern 46 | *Pyracantha fortuneana* (Maxim.) H.L.Li (Rosaceae) | 13227 | OQ615655 |
|  |  |  | *Aculus* sp. | Z316 | ATA | Pattern 46 | *Sapindus saponaria* L. (Sapindaceae) | 13525 | OQ615715 |
|  |  |  | *Aculus* sp. | GZ134 | ATA | Pattern 46 | *Wikstroemia nutans* Champ. ex Benth. (Thymelaeaceae) | 13541 | OQ615647 |
|  |  |  | *Tegolophus alangii* | Z296 | ATA | Pattern 46 | *Alangium chinense* (Lour.) Harms (Alangiaceae) | 13374 | OQ615709 |
|  |  |  | *Tegolophus celtis* | S136 | ATA | Pattern 48 | *Celtis sinensis* Pers. (Cannabaceae) | 13830 | OQ615687 |
|  |  |  | *Tegolophus fargesiae* | E1 | ATA | Pattern 46 | *Lespedeza* sp. (Fabaceae) | 14261 | OQ615614 |
|  |  |  | *Tegolophus hunanensis* | AH39 | ATA | Pattern 46 | *Vitex negundo* L. (Lamiaceae) | 13895 | OQ615592 |
|  |  |  | *Tegolophus hunanensis* | S152 | ATA | Pattern 46 | *Vitex agnus-castus* L. (Lamiaceae) | 13757 | OQ615691 |
|  |  |  | *Tegolophus hunanensis* | HEN17 | ATA | Pattern 46 | *Vitex negundo* L. (Lamiaceae) | 13745 | OQ615656 |
|  |  |  | *Tegolophus hunanensis* | HEN41 | ATA | Pattern 46 | *Vitex negundo* L. (Lamiaceae) | 13747 | OQ615665 |
|  |  |  | *Tegolophus lespedezae* | HEN12 | ATA | Pattern 46 | *Lespedeza bicolor*Turcz. (Fabaceae) | 13445 | OQ615652 |
|  |  |  | *Tegolophus miscanthus* | Z336 | ATA | Pattern 46 | *Miscanthus floridulus* (Labill.) Warb. ex K.Schum. & Lauterb. (Poaceae) | 13491 | OQ615721 |
|  |  |  | *Tegolophus* c.f. *miscanthus* | Z371 | RCP | Pattern 35 | *Miscanthus floridulus* (Labill.) Warb. ex K.Schum. & Lauterb. (Poaceae) | 13922 | OQ615730 |
|  |  |  | *Tegolophus oblatus* | QH7 | ATA | Pattern 46 | *Syzygium aromaticum* (L.) Merr. & L. M. Perry (Oleaceae) | 13475 | OQ615685 |
|  |  |  | *Tegolophus ringsi* | CA37 | RCP | Pattern 27 | *Celtis occidentalis* L. (Cannabaceae) | 13893 | OQ615605 |
|  |  |  | *Tegolophus* sp. | CA38 | RCP | Pattern 27 | *Celtis tenuifolia* Nutt. (Cannabaceae) | 13896 | OQ615606 |
|  |  |  | *Tegolophus suffruticosae* | LN14 | RCP | Pattern 41 | *Flueggea suffruticosa* (Pall.) Baill. (Euphorbiaceae) | 13543 | OQ615681 |
|  |  |  | *Tegolophus suffruticosae* | Z382 | RCP | Pattern 40 | *Triadica cochinchinensis* Lour. (Euphorbiaceae) | 13469 | OQ615733 |
|  |  |  | *Tegolophus tremae* | Z368 | ATA | Pattern 46 | *Trema cannabina* Lour. (Ulmaceae) | 13576 | OQ615729 |
|  |  |  | *Tegolophus ulmi* | CQ4 | ATA | Pattern 46 | *Ulmus pumila* L. (Ulmaceae) | 13417 | OQ615613 |
|  |  |  | *Tegolophus* sp. | HK8 | ATA | Pattern 46 | *Celtis* sp. (Cannabaceae) | 13738 | OQ615670 |
|  |  |  | *Tetra cuihuae* | QH6 | ATA | Pattern 46 | *Populus przewalskii* Maxim. (Salicaceae) | 13409 | OQ615684 |
|  |  |  | *Tetra louguantais* | AH43 | ATA | Pattern 46 | *Pistacia chinensis* Bunge (Anacardiaceae) | 13605 | OQ615596 |
|  |  |  | *Tetra lycopersici* | FQ | ATA | Pattern 50 | *Solanum lycopersicum* L. (Solanaceae) | 13865 | OQ615620 |
|  |  |  | *Tetra lucidi* | HEN8 | ATA | Pattern 46 | *Ligustrum quihoui* Carrière (Oleaceae) | 13481 | OQ615651 |
|  |  |  | *Tetra pyriana* | HLJ98 | ATA | Pattern 46 | *Pyrus calleryana* Decne. (Rosaceae) | 13454 | OQ615672 |
|  |  |  | *Tetra qingyuanensis* | Z333 | ATA | Pattern 46 | *Lespedeza bicolor*Turcz. (Fabaceae) | 13436 | OQ615720 |
|  |  |  | *Tetra suspensae* | SD22 | ATA | Pattern 46 | *Forsythia suspensa* (Thunb.) Vahl (Oleaceae) | 13506 | OQ615698 |
|  |  |  | *Tetra* sp. | SD27 | ATA | Pattern 46 | *Rottboellia cochinchinensis* (Lour.) Clayton (Poaceae) | 13470 | OQ615701 |
|  |  |  | *Tetra* sp. | Z400 | ATA | Pattern 46 | *Callerya tsui* (F.P. Metcalf) Z. Wei & Pedley (Fabaceae) | 13425 | OQ615738 |
|  |  |  | *Tetra* sp. | CA43 | ATA | Pattern 46 | *Rhamnus cathartica* L. (Rhamnaceae) | 13501 | OQ615609 |
|  |  |  | *Tetra* sp. | Z284 | ATA | Pattern 46 | *Phaenosperma globosum* Munro ex Oliv. (Poaceae) | 13509 | OQ615706 |
|  |  |  | *Tetra* sp. | GZ35 | ATA | Pattern 50 | *Trema cannabina* Lour. (Cannabaceae) | 13527 | OQ615637 |
|  |  |  | *Tetraspinus* sp. | HEN26 | ATA | Pattern 46 | *Nageia nagi* (Thunb.) Kuntze (Podocarpaceae ) | 13593 | OQ615660 |
|  |  |  | *Vittacus cannabus* | HEN44 | ATA | Pattern 46 | *Cannabis sativa* L. (Cannabaceae) | 13737 | OQ615666 |
|  |  | Phyllocoptini | *Calepitrimerus anomalus* | HLJ99 | “*Calepitrimerus s.l.*” | Pattern 53 | *Paeonia lactiflora* Pall. (Paeoniaceae) | 13645 | OQ615673 |
|  |  |  | *Calepitrimerus flexuosus* | Z342 | “*Calepitrimerus s.l.*” | Pattern 54 | *Spiraea fritschiana* C.K.Schneid. (Rosaceae) | 13652 | OQ615724 |
|  |  |  | *Calepitrimerus* sp. | CA2 | “*Calepitrimerus s.l.*” | Pattern 54 | *Rhus typhina* L. (Anacardiaceae) | 13650 | OQ615598 |
|  |  |  | *Calepitrimerus bungeanus* | HLJ97 | ATA | Pattern 46 | *Euonymus maackii* Rupr. (Celastraceae) | 13401 | OQ615671 |
|  |  |  | *Calepitrimerus* sp. | Z308 | RCP | Pattern 29 | *Sassafras tzumu* (Hemsl.) Hemsl. (Lauraceae) | 13918 | OQ615714 |
|  |  |  | *Cereusacarus* sp. | Z361 | ATA | Pattern 46 | *Symplocos paniculata* (Thunb.) Miq. (Symplocaceae ) | 13981 | OQ615727 |
|  |  |  | *Epitrimerus sabinae* | GZ121 | RCP | Pattern 33 | *Cryptomeria japonica* (Thunb. ex L.f.) D.Don (Cupressaceae) | 13757 | OQ615644 |
|  |  |  | *Epitrimerus* c.f. *sabinae* | Xue et al. [35] | RCP | Pattern 26 | *Juniperus chinensis* L. (Cupressaceae) | 13531 | NC029208 |
|  |  |  | *Epitrimerus* c.f. *sabinae* | HEN25 | RCP | Pattern 26 | *Juniperus chinensis* var. *aurea* M.Young (Cupressaceae) | 13618 | OQ615659 |
|  |  |  | *Epitrimerus* c.f. *sabinae* | Z300 | RCP | Pattern 33 | *Cryptomeria japonica* (Thunb. ex L.f.) D.Don (Cupressaceae) | 13847 | OQ615710 |
|  |  |  | *Epitrimerus* c.f. *sabinae* | Z344 | RCP | Pattern 33 | *Cryptomeria japonica* (Thunb. ex L.f.) D.Don (Cupressaceae) | 14289 | OQ615726 |
|  |  |  | *Epitrimerus* c.f. *sabinae* | Z343 | RCP | Pattern 31 | *Fokienia hodginsii* (Dunn) A.Henry & H H.Thomas (Cupressaceae) | 14438 | OQ615725 |
|  |  |  | *Epitrimerus* c.f. *spirae* | KK1 | ATA | Pattern 51 | *Cryptomeria* sp. (Cupressaceae) | 13638 | OQ615678 |
|  |  |  | *Epitrimerus pyri* | AH40 | “*Calepitrimerus s.l.*” | Pattern 52 | *Pyrus* sp. (Rosaceae) | 13773 | OQ615593 |
|  |  |  | *Epitrimerus* sp. | Z338 | ATA | Pattern 46 | *Callerya nitida* (Benth.) R.Geesink (Fabaceae) | 13425 | OQ615722 |
|  |  |  | *Epitrimerus* sp. | SD25 | RCP | Pattern 18 | *Bistorta officinalis* Delarbre (Polygonaceae) | 13590 | OQ615700 |
|  |  |  | *Epitrimerus* sp. | S153 | RCP | Pattern 24 | *Phoebe hunanensis* Hand-Mazz. (Lauraceae) | 13739 | OQ615692 |
|  |  |  | *Fujianacarus wisterianis* | Z399 | RCP | Pattern 34 | *Callerya nitida* (Benth.) R.Geesink (Fabaceae) | 13462 | OQ615737 |
|  |  |  | *Fujianacarus wisterianis* | HN237 | RCP | Pattern 34 | *Callerya nitida* (Benth.) R.Geesink (Fabaceae) | 14210 | OQ615675 |
|  |  |  | *Fujianacarus wisterianis* | CQ1 | RCP | Pattern 34 | *Spatholobus suberectus* Dunn (Fabaceae) | 13485 | OQ615612 |
|  |  |  | *Grandiscapitis camelliae* | YN453 | RCP | Pattern 36 | *Camellia oleifera* Abel (Theaceae) | 13503 | OQ615703 |
|  |  |  | *Keiferella guanegouensis* | QH1 | RCP | Pattern 22 | *Picea asperata* Mast. (Pinaceae) | 13972 | OQ615682 |
|  |  |  | *Leipothrix* c.f. *sabinae* | HEN4 | RCP | Pattern 24 | *Platycladus orientalis* (L.) Franco (Cupressaceae) | 13460 | OQ615650 |
|  |  |  | *Leipothrix* sp. | Z372 | RCP | Pattern 18 | *Symplocos lancifolia* Sieb & Zucc. (Symplocaceae) | 13806 | OQ615731 |
|  |  |  | *Leipothrix* sp. | Xue et al. [36] | RCP | Pattern 32 | *Juniperus chinensis* L. (Cupressaceae) | 14216 | KX027362 |
|  |  |  | *Neocalepitrimerus rosa* | GX37 | RCP | Pattern 44 | *Rosa multiflora* Thunb. (Rosaceae) | 13876 | OQ615634 |
|  |  |  | *Neoepitrimerus platycladi* | S138_1 | RCP | Pattern 24 | *Platycladus orientalis* (L.) Franco (Cupressaceae) | 13452 | OQ615688 |
|  |  |  | *Paraepitrimerus* sp. | H93 | ATA | Pattern 50 | *Paeonia* sp. (Paeoniaceae) | 13430 | OQ615648 |
|  |  |  | *Phyllocoptruta platyclada* | S138_2 | RCP | Pattern 24 | *Platycladus orientalis* (L.) Franco (Cupressaceae) | 13531 | OQ615689 |
|  |  |  | *Phyllocoptruta platyclada* | G6 | RCP | Pattern 26 | *Platycladus orientalis* (L.) Franco (Cupressaceae) | 13558 | OQ615621 |
|  |  |  | *Phyllocoptruta* sp. | CA6 | ATA | Pattern 46 | *Thuja occidentalis* L. (Cupressaceae) | 14083 | OQ615599 |
|  |  |  | *Phyllocoptes taishanensis* | Xue et al. [35] | RCP | Pattern 23 | *Cedrus deodara* (Roxb. ex D. Don) G. Don (Pinaceae) | 13496 | NC029209 |
|  |  |  | *Phyllocoptes* c.f. *taishanensis* | S167 | RCP | Pattern 25 | *Metasequoia glyptostroboides* Hu & W. C. Cheng (Cupressaceae) | 14073 | OQ615693 |
|  |  |  | *Prominens photiniae* | FJ26 | RCP | Pattern 44 | *Photinia serratifolia* (Desf.) Kalkman (Rosaceae) | 14115 | OQ615618 |
|  |  |  | *Proiectus thunbergis* | HEN28 | ATA | Pattern 46 | *Pinus thunbergii* Parl. (Pinaceae) | 13563 | OQ615661 |
|  |  |  | *Proiectus granularpro* | Z304 | RCP | Pattern 23 | *Pinus* sp. (Pinaceae) | 13899 | OQ615713 |
|  |  |  | *Vasates* sp. | Z327 | ATA | Pattern 46 | *Archidendron lucidum* (Benth) I. C. Nielsen (Fabaceae) | 13432 | OQ615718 |
|  |  | Tegonotini | *Shevtchenkella miscanthis* | KK11 | ATA | Pattern 46 | *Miscanthus floridulus* (Labill.) Warb. ex K.Schum. & Lauterb. (Poaceae) | 13601 | OQ615679 |
|  |  |  | *Scolotosus* sp. | GX54 | ATA | Pattern 46 | *Ehretia acuminata* R. Br. (Boraginaceae) | 13702 | OQ615635 |
|  |  |  | *Scolotosus* sp. | GZ100 | ATA | Pattern 46 | *Ehretia* sp. (Boraginaceae) | 13610 | OQ615643 |
|  |  |  | *Tegonotus celtis* | YN448 | ATA | Pattern 46 | *Celtis sinensis* Pers. (Cannabaceae) | 13437 | OQ615702 |
| Diptilomiopidae | Diptilomiopinae |  | *Acarhis* sp. | Z277 | Diptilomiopinae | Pattern 11 | *Machilus nanmu* (Oliv.) Hemsl. (Lauraceae) | 13572 | OQ615705 |
|  |  |  | *Apodiptacus castaneae* | Z377 | Diptilomiopinae | Pattern 17 | *Castanea seguinii* Dode (Fagaceae) | 14269 | OQ615732 |
|  |  |  | *Apodiptacus toxicodendri* | FJ25 | Diptilomiopinae | Pattern 10 | *Toxicodendron vernicifluum* (Stokes) F. A. Barkley. (Anacardiaceae) | 13843 | OQ615617 |
|  |  |  | *Apodiptacus* sp. | E11 | Diptilomiopinae | Pattern 8 | *Castanea mollissima* Blume (Fagaceae) | 13906 | OQ615615 |
|  |  |  | *Diptacus brevichaetus* | Z365 | Diptilomiopinae | Pattern 16 | *Lindera communis* Hemsl. (Lauraceae) | 14408 | OQ615728 |
|  |  |  | *Diptacus* sp. | CA46 | Diptilomiopinae | Pattern 14 | *Juglans nigra* L. (Juglandaceae) | 13773 | OQ615610 |
|  |  |  | Diptilomiopinae sp. | GZ126 | Diptilomiopinae | Pattern 12 | *Glochidion wilsonii* Hutch. (Phyllanthaceae) | 13812 | OQ615646 |
|  |  |  | Diptilomiopinae sp. | GZ38 | Diptilomiopinae | Pattern 10 | *Ligustrum lucidum* W.T.Aiton (Oleaceae) | 14065 | OQ615638 |
|  |  |  | *Diptilomiopus* sp. | Z388 | Diptilomiopinae | Pattern 15 | *Phoebe sheareri* (Hemsl.) Gamble (Lauraceae) | 13904 | OQ615734 |
|  |  |  | *Diptilomiopus* sp. | LN6 | Diptilomiopinae | Pattern 13 | *Celtis koraiensis* Nakai (Cannabaceae) | 13770 | OQ615680 |
|  |  |  | *Trimeroptes luanchuanensis* | Z322 | Diptilomiopinae | Pattern 9 | *Broussonetia kazinoki* Siebold (Moraceae) | 13925 | OQ615717 |
|  | Rhyncaphytoptinae |  | *Quadracus cudraniae* | AH41 | RCP | Pattern 18 | *Maclura tricuspidata* Carrière (Moraceae) | 13766 | OQ615594 |
|  |  |  | *Rhinotergum shaoguanense* | Xue et al. [36] | RCP | Pattern 18 | *Ulmus pumila* L. (Ulmaceae) | 13646 | NC034150 |
|  |  |  | *Rhinophytoptus broussonetiae* | GZ98 | RCP | Pattern 19 | *Miscanthus floridulus* (Labill.) Warb. ex K.Schum. & Lauterb. (Poaceae) | 13615 | OQ615642 |
|  |  |  | *Rhinophytoptus broussonetiae* | GZ74 | RCP | Pattern 20 | *Broussonetia kazinoki* Siebold (Moraceae) | 13711 | OQ615639 |
|  |  |  | *Rhinophytoptus broussonetiae* | Z401 | RCP | Pattern 19 | *Broussonetia kazinoki* Siebold (Moraceae) | 13314 | OQ615739 |
|  |  |  | *Rhinophytoptus* c.f. *broussonetiae* | Z292 | RCP | Pattern 21 | *Broussonetia kazinoki* Siebold (Moraceae) | 13496 | OQ615708 |
|  |  |  | *Rhinophytoptus* c.f. *sericeaomeiensis* | AH42 | RCP | Pattern 18 | *Celtis sinensis* Pers. (Cannabaceae) | 13653 | OQ615595 |
|  |  |  | *Rhyncaphytoptus redwayensis* | CA28 | RCP | Pattern 18 | *Quercus macrocarpa* Michx. (Fagaceae) | 13765 | OQ615603 |
|  |  |  | *Rhyncaphytoptus ulmi* | HEN3 | RCP | Pattern 18 | *Ulmus pumila* L. (Ulmaceae) | 13980 | OQ615649 |
|  |  |  | *Rhyncaphytoptus* sp. | GS9 | RCP | Pattern 18 | *Populus euphratica* Oliv. (Salicaceae) | 13667 | OQ615633 |
|  |  |  | *Rhyncaphytoptus* sp. | GZ96 | RCP | Pattern 20 | *Quercus aliena* Blume (Fagaceae) | 13904 | OQ615641 |

RCP: Rhyncaphytoptinae + Cecidophyinae + “Phyllocoptini” ; ATA: “Anthocoptini” + Tegonotini + Aceriini

Additional file 1: Table S2. Breakpoints of eriophyoid mitochondrial genomes with different gene arrangement patterns

| **Pattern** | **breakpoints** | **Family** | **Species Number** |
| --- | --- | --- | --- |
| Pattern 1 | 18 | Phytoptidae | 3 |
| Pattern 2 | 16 | Phytoptidae | 1 |
| Pattern 3 | 11 | Phytoptidae | 1 |
| Pattern 4 | 17 | Eriophyidae | 2 |
| Pattern 5 | 18 | Eriophyidae | 1 |
| Pattern 6 | 18 | Eriophyidae | 1 |
| Pattern 7 | 19 | Eriophyidae | 1 |
| Pattern 8 | 21 | Diptilomiopidae | 1 |
| Pattern 9 | 22 | Diptilomiopidae | 1 |
| Pattern 10 | 23 | Diptilomiopidae | 2 |
| Pattern 11 | 21 | Diptilomiopidae | 1 |
| Pattern 12 | 20 | Diptilomiopidae | 1 |
| Pattern 13 | 22 | Diptilomiopidae | 1 |
| Pattern 14 | 20 | Diptilomiopidae | 1 |
| Pattern 15 | 23 | Diptilomiopidae | 1 |
| Pattern 16 | 22 | Diptilomiopidae | 1 |
| Pattern 17 | 20 | Diptilomiopidae | 1 |
| Pattern 18 | 15 | Diptilomiopidae | 8 |
| Pattern 19 | 15 | Diptilomiopidae | 3 |
| Pattern 20 | 14 | Diptilomiopidae | 1 |
| Pattern 21 | 17 | Diptilomiopidae | 1 |
| Pattern 22 | 15 | Eriophyidae | 1 |
| Pattern 23 | 13 | Eriophyidae | 3 |
| Pattern 24 | 15 | Eriophyidae | 3 |
| Pattern 25 | 13 | Eriophyidae | 1 |
| Pattern 26 | 16 | Eriophyidae | 4 |
| Pattern 27 | 20 | Eriophyidae | 2 |
| Pattern 28 | 15 | Eriophyidae | 1 |
| Pattern 29 | 15 | Eriophyidae | 1 |
| Pattern 30 | 15 | Eriophyidae | 1 |
| Pattern 31 | 18 | Eriophyidae | 1 |
| Pattern 32 | 16 | Eriophyidae | 1 |
| Pattern 33 | 19 | Eriophyidae | 3 |
| Pattern 34 | 20 | Eriophyidae | 3 |
| Pattern 35 | 15 | Eriophyidae | 1 |
| Pattern 36 | 14 | Eriophyidae | 1 |
| Pattern 37 | 15 | Eriophyidae | 1 |
| Pattern 38 | 15 | Eriophyidae | 1 |
| Pattern 39 | 17 | Eriophyidae | 1 |
| Pattern 40 | 17 | Eriophyidae | 1 |
| Pattern 41 | 17 | Eriophyidae | 1 |
| Pattern 42 | 25 | Eriophyidae | 1 |
| Pattern 43 | 15 | Eriophyidae | 1 |
| Pattern 44 | 15 | Eriophyidae | 2 |
| Pattern 45 | 15 | Eriophyidae | 1 |
| Pattern 46 | 18 | Eriophyidae | 69 |
| Pattern 47 | 21 | Eriophyidae | 3 |
| Pattern 48 | 18 | Eriophyidae | 1 |
| Pattern 49 | 19 | Eriophyidae | 1 |
| Pattern 50 | 21 | Eriophyidae | 3 |
| Pattern 51 | 18 | Eriophyidae | 1 |
| Pattern 52 | 19 | Eriophyidae | 1 |
| Pattern 53 | 18 | Eriophyidae | 1 |
| Pattern 54 | 21 | Eriophyidae | 2 |

Breakpoints were calculated with CREx as a measure of the extent of mt gene rearrangement relative to that of the hypothetical ancestor of arthropods.

Additional file 1: Table S3. Outgroups used in phylogenetic analysis based on mitochondrial genome sequences

| Superorder | Order | Superfamily | Family | Species | Size (bp) | GenBank number | Reference |
| --- | --- | --- | --- | --- | --- | --- | --- |
|  | Pantopoda |  | Nymphonidae | *Nymphon gracile* | 14681 | DQ666063 | [55] |
|  |  |  | Ammotheidae | *Ammothea carolinensis* | 15101 | GU065293 | [56] |
|  |  |  |  | *Achelia bituberculata* | 15474 | AY457170 | [57] |
|  |  |  | Tanystylidae | *Tanystylum orbiculare* | 15251 | GU370074 | [27] |
|  | Xiphosura |  | Limulidae | *Carcinoscorpius rotundicauda* | 15033 | JQ178358 | [58] |
|  |  |  |  | *Limulus polyphemus* | 14985 | NC003057 | [59] |
|  |  |  |  | *Tachypleus tridentatus* | 15008 | NC012574 | [58] |
|  | Solifugae |  | Eremobatidae | *Eremobates c.f palpisetulosus* | 15083 | EU520642 | [60] |
|  |  |  | Ammotrechidae | *Nothopuga* sp. | 14984 | NC009984 | [61] |
|  | Thelyphonida |  | Thelyphonidae | *Mastigoproctus giganteus* | 14416 | EU520643 | [60] |
|  | Amblypygi |  | Phrynidae | *Phrynus* sp. | 14764 | EU520641 | [60] |
|  |  |  | Phrynichidae | *Damon diadema* | 14786 | NC011293 | [62] |
|  | Scorpiones |  | Scorpionidae | *Heterometrus longimanus* | 14655 | NC029207 | [63] |
|  |  |  | Buthidae | *Tityus serrulatus* | 14460 | NC027855 | [64] |
|  |  |  |  | *Mesobuthus martensii* | 15034 | NC009738 | [63] |
|  | Araneae |  | Liphistiidae | *Liphistius erawan* | 14197 | NC020323 | [65] |
|  |  |  | Theraphosidae | *Ornithoctonus huwena* | 13874 | NC005925 | [66] |
|  |  |  | Pholcidae | *Pholcus phalangioides* | 14459 | KJ782458 | [67] |
|  |  |  | Cybaeidae | *Argyroneta aquatica* | 16000 | NC026863 | [68] |
|  |  |  | Pelleninae | *Habronattus oregonensis* | 14381 | NC005942 | [69] |
|  |  |  |  | *Neoscona theisi* | 14156 | NC026290 | [70] |
|  | Opiliones |  | Phalangiidae | *Oligolophus tienmushanensis* | 16798 | NC029887 | [63] |
|  |  |  |  | *Opilio parietinus* | 15400 | NC014700 | [71] |
|  |  |  |  | *Phalangium opilio* | 14968 | EU523757 | [60] |
|  | Ricinulei |  | Ricinoididae | *Pseudocellus gertschi* | 14477 | NC023451 | unpublished |
|  |  |  |  | *Pseudocellus pearsei* | 15099 | EU024483 | [61] |
|  |  |  |  | *Cryptocellus narino* | 14554 | NC023450 | unpublished |
|  |  |  |  | *Ricinoides karschii* | 14614 | NC023452 | unpublished |
|  | Pseudoscorpiones |  | Pseudogarypidae | *Pseudogarypus banksi* | 16546 | JQ040544 | [72] |
|  |  |  | Atemnidae | *Paratemnoides elongatus* | 14368 | NC017752 | [72] |
| Parasitiformes | Mesostigmata | Cercomegistoidea | Asternoseiidae | *Asternoseius* sp. | 14655 | MN622221 | [29] |
|  |  | Celaenopsoidea | Diplogyniidae | *Trematuridae* sp. | 14997 | MN622242 | [29] |
|  |  | Rhodacaroidea | Rhodacaridae | *Rhodacarus* sp. | 14761 | MN622243 | [29] |
|  |  | Ascoidea | Ologamasidae | *Stylochyrus rarior* | 14900 | NC013474 | [29] |
|  |  |  |  | *Pergamasus crassipes* | 19144 | MN622231 | [29] |
|  |  | Phytoseioidea | Phytoseiidae | *Euseius nicholsi* | 15524 | NC026788 | [73] |
|  |  | Dermanyssoidea | Laelapidae | *Gaeolaelaps aculeifer* | 15130 | MN622222 | [29] |
|  | Holothyrida | Holothyroidea | Allothyridae | *Allothyrus* sp*.* | 14578 | KC769586 | [74] |
|  | Ixodida | Ixodoidea | Argasidae | *Argas lagenoplastis* | 14478 | NC023369 | [74] |
|  |  |  |  | *Ornithodoros brasiliensis* | 14489 | NC023373 | [74] |
|  |  |  |  | *Nothoaspis amazoniensis* | 14416 | NC033900 | [75] |
|  |  |  | Ixodidae | *Ixodes holocyclus* | 15007 | NC005293 | [76] |
|  |  |  |  | *Robertsicus elaphensis* | 14627 | NC017758 | [77] |
|  |  |  |  | *Haemaphysalis bancrofti* | 14673 | NC041076 | [78] |
|  |  |  |  | *Dermacentor silvarum* | 14945 | NC026552 | [79] |
|  |  |  |  | *Amblyomma cajennense* | 14780 | NC020333 | [80] |
|  |  |  |  | *Rhipicephalus geigyi* | 14948 | NC023350 | [74] |
| Acariformes | Sarcoptiformes | Pterolichoidea | Pterolichidae | *Ardeacarus ardeae* | 14069 | KY352304 | [81] |
|  |  | Epilohmannioidea | Epilohmanniidae | *Epilohmannia* sp. | 14050 | MN622165 | [29] |
|  |  | Phthiracaroidea | Steganacaridae | *Steganacarus magnus* | 13818 | NC011574 | [82] |
|  |  | Sarcoptoidea | Sarcoptidae | *Sarcoptes scabiei* | 13837 | AP017940 | [83] |
|  |  | Analgoidea | Pyroglyphidae | *Dermatophagoides farinae* | 14266 | NC013184 | [84] |
|  |  | Histiostomatoidea | Histiostomatidae | *Histiostoma blomquisti* | 15892 | NC031377 | [85] |
|  |  | Acaroidea | Acaridae | *Acarus farris* | 13874 | MZ425442 | [30] |
|  |  |  |  | *Rhizoglyphus robini* | 14244 | NC038058 | [86] |
|  |  | Hermannielloidea | Hermanniellidae | *Hermanniella granulata* | 14549 | MN622192 | [29] |
|  |  | Gustavioidea | Ceratoppiidae | *Ceratoppia bipilis* | 14763 | MN622161 | [29] |
|  |  |  | Xenillidae | *Xenillus tegeocranus* | 14806 | MN622216 | [29] |
|  |  | Oppioidea | Oppiidae | *Oppia africana* | 13932 | MN622234 | [29] |
|  |  | Licneremaeoidea | Scutoverticidae | *Bipassalozetes perforatus* | 13836 | MN622229 | [29] |
|  |  | Phenopelopoidea | Humerobatidae | *Humerobates rostrolamellatus* | 14060 | MN622230 | [29] |
|  |  | Oripodoidea | Oribatulidae | *Zygoribatula* sp. | 14880 | MN622179 | [29] |
|  |  |  |  | *Oribatula tibialis* | 14543 | MN622223 | [29] |
|  |  |  | Scheloribatidae | *Paraleius leontonychus* | 14186 | LT984407 | [87] |
|  | Trombidiformes | Pygmephoroidea | Pygmephoridae | *Mahunkania secundademo* | 14102 | MZ425440 | [30] |
|  |  |  |  | *Demodex folliculorum* | 14150 | NC026102 | [88] |
|  |  | Tetranychoidea | Tetranychidae | *Oligonychus shinkajii* | 13097 | MZ425445 | [30] |
|  |  |  |  | *Panonychus ulmi* | 13115 | NC012571 | [89] |
|  |  |  |  | *Amphitetranychus* *viennensis* | 13085 | NC032381 | unpublished |
|  |  |  |  | *Tetranychus urticae* | 13103 | NC024677 | [90] |
|  |  | Sperchontoidea | Sperchontidae | *Sperchon plumifer* | 14646 | NC039813 | [89] |
|  |  | Trombiculoidea | Trombiculidae | *Walchia hayashii* | 14856 | NC010595 | unpublished |
|  |  |  |  | *Leptotrombidium deliense* | 13731 | NC007600 | [91] |
|  |  |  |  | *Trombiculidae* sp. | 14207 | MN622164 | [29] |

Additional file 1: Table S4. Sequence of primers used in this study

| **Primer Tag** | **Primer sequence** | **Primer length(bp)** |
| --- | --- | --- |
| PM-C1-R1 | CCYCGTCGDTAT*T*C | 14 |
| PM-C1-R2 | TGAAARTGRGCT*A*C | 14 |
| PM-N10-C1-L | GGTCAYCCTGA*G*G | 13 |
| PM-N54-C1-L | CCYAAAGGRTCA*A*A | 14 |
| PM-N53-C1-L | CAVCCDGGT*T*C | 11 |
| PM-N4-C1-H | CCAATRTCYTTA*T*G | 14 |
| PM-N11-C2-H | ATGAYAACTTGA*A*A | 14 |
| PM-C2-R1 | TCAATAYCAT*T*G | 12 |
| PM-C2-L1 | GATGTWCTTCAT*T*C | 14 |
| PM-C2-L2 | GATTAGCYCC*A*C | 12 |
| PM-N17-C2-L | GBTTTATRCCTAT*T*G | 15 |
| PM-A6-L2 | GAATHAAAGAT*A*C | 13 |
| PM-C3-L1 | TTYATTTCTTCT*G*A | 14 |
| PM-C3-R1 | TCNACRAAATGT*C*A | 14 |
| PM-N5-N3-H | GAATGTGGDTTT*A*C | 14 |
| PM-N5-R2 | GCWTTYTCTACT*A*T | 14 |
| PM-N5-R1 | YATCHCCTAAT*C*G | 13 |
| PM-O46-N4-H | CAYGGCTTGRTC*T*C | 14 |
| PM-N4-L1 | GAGCTTTGGTHAG*C*C | 15 |
| PM-O39-CB-L | GGAGCCAAAATTT*C*A | 15 |
| PM-CB-R1 | CGATTYTACTCT*T*T | 14 |
| PM-N15-CB-L | CTGGTTGAATAT*G*A | 14 |
| PM-N1-R1 | ACTGCYTCATAA*G*A | 14 |
| PM-N1-L1 | CCTATWASAGAT*G*C | 14 |
| PM-N12-N1-L | GTCAAAAAAGCA*A*C | 14 |
| PM-O51-12S-H | CCGCGKCKGCTG*G*C | 14 |
| PM-12S-R1 | GGATTAGATAC*C*C | 13 |
| PM-O50-12S-L | TACTTTGTTACG*A*C | 14 |
| PM-O42-16S-H | CCTYTTGTATT*A*G | 13 |
| PM-O41-16S-L | CTGAACTCAGA*T*C | 13 |
| PM-N2-R2 | GGTATNTTCCCC*T*T | 14 |

*Phosphorthioate

Additional file 1: Table S5. Datasets used in phylogenetic analysis through different partitions

| **Data Type** | **length (bp)** | **Partition Type** | | **Partition**  **Number** | **Tree-building**  **Method** |
| --- | --- | --- | --- | --- | --- |
| Nucleotide | 10071 | gene | 15 | | MrBayes |
|  |  |  |  |  | ML_GTRGAMMAI |
|  |  |  |  |  | ML_GTRCAT |
|  |  | codon | 3 | | MrBayes |
|  |  |  |  |  | ML_GTRGAMMAI |
|  |  |  |  |  | ML_GTRCAT |
|  |  | PartitionFinder | 6 | | MrBayes |
|  |  |  |  |  | ML_GTRGAMMAI |
|  |  |  |  |  | ML_GTRCAT |
|  | 7153 | gene  (excluding third codon positions) | 13 | | MrBayes |
|  |  |  |  |  | ML_GTRGAMMAI |
|  |  |  |  |  | ML_GTRCAT |
|  |  | codon (excluding third codon positions) | 2 | | MrBayes |
|  |  |  |  |  | ML_GTRGAMMAI |
|  |  |  |  |  | ML_GTRCAT |
| Amino Acid | 2268 | gene | 13 | | MrBayes |
|  |  |  |  |  | ML_ProteinGAMMA |
|  |  |  |  |  | ML_ProteinCAT |
|  |  | PartitionFinder | 3 | | MrBayes |
|  |  |  |  |  | ML_ProteinGAMMA |
|  |  |  |  |  | ML_ProteinCAT |
|  |  | no partition | 1 | | IQtree2-PMSF |

Additional file 1: Table S6. Arachnida species included in this study in correlation analysis between rate of gene rearrangement and rate of nucleotide substitution

| **Taxon** | **Species** | **Breakpoints** | **Ka** | **GenBank number** |
| --- | --- | --- | --- | --- |
| Parasitiformes | *Robertsicus elaphensis* | 7 | 0.3027 | NC017758 |
|  | *Amblyomma cajennense* | 7 | 0.2967 | NC020333 |
|  | *Pergamasus crassipes* | 3 | 0.2753 | MN622231 |
|  | *Stylochyrus rarior* | 5 | 0.2768 | NC013474 |
|  | *Pergamasus* sp. | 0 | 0.2526 | MN622219 |
|  | *Trematuridae* sp. | 8 | 0.2663 | MN622242 |
|  | *Asternoseius* sp. | 3 | 0.2524 | MN622221 |
|  | *Parasitidae* sp. | 0 | 0.2360 | MN622232 |
|  | *Euseius nicholsi* | 30 | 0.4159 | NC026788 |
|  | *Gaeolaelaps aculeifer* | 15 | 0.2880 | MN622222 |
|  | *Ixodes holocyclus* | 0 | 0.2451 | NC005293 |
|  | *Amblyomma triguttatum* | 7 | 0.2817 | NC005963 |
|  | *Haemaphysalis bancrofti* | 7 | 0.2936 | NC041076 |
|  | *Dermacentor silvarum* | 7 | 0.3013 | NC026552 |
|  | *Rhipicephalus geigyi* | 7 | 0.2927 | NC023350 |
|  | *Rhodacarus* sp. | 0 | 0.2618 | MN622243 |
|  | *Allothyrus* sp. | 0 | 0.2198 | KC769586 |
|  | *Ornithodoros moubata* | 0 | 0.2469 | NC004357 |
|  | *Argas lagenoplastis* | 0 | 0.2325 | NC023369 |
|  | *Nothoaspis amazoniensis* | 0 | 0.2465 | NC033900 |
|  | *Ornithodoros brasiliensis* | 0 | 0.2405 | NC023373 |
| Ricinulei | *Ricinoides karschii* | 0 | 0.2470 | NC023452 |
|  | *Cryptocellus narino* | 3 | 0.2315 | NC023450 |
|  | *Pseudocellus gertschi* | 0 | 0.2532 | NC023451 |
|  | *Pseudocellus pearsei* | 11 | 0.2398 | EU024483 |
| Solifugae | *Eremobates* c.f. *palpisetulosus* | 2 | 0.1901 | EU520642 |
|  | *Nothopuga* sp. | 3 | 0.1914 | NC009984 |
| Xiphosura | *Carcinoscorpius rotundicauda* | 0 | 0.0790 | JQ178358 |
|  | *Tachypleus tridentatus* | 3 | 0.0741 | NC012574 |
|  | *Limulus polyphemus* | 0 | 0.0000 | NC003057 |
| Amblypygi | *Damon diadema* | 0 | 0.2397 | NC011293 |
|  | *Phrynus* sp. | 0 | 0.2289 | EU520641 |
| Thelyphonida | *Mastigoproctus giganteus* | 3 | 0.2499 | EU520643 |
| Opiliones | *Phalangium opilio* | 5 | 0.2253 | EU523757 |
|  | *Oligolophus tienmushanensis* | 4 | 0.2307 | NC029887 |
|  | *Opilio parietinus* | 4 | 0.2304 | NC014700 |
| Araneae | *Selenops bursarius* | 16 | 0.3222 | NC024878 |
|  | *Agelena silvatica* | 17 | 0.3299 | NC033971 |
|  | *Araneus angulatus* | 16 | 0.3273 | NC032402 |
|  | *Calisoga longitarsis* | 15 | 0.3688 | EU523754 |
|  | *Phyxioschema suthepium* | 15 | 0.3700 | NC020322 |
|  | *Oxytate striatipes* | 16 | 0.3258 | NC025557 |
|  | *Habronattus oregonensis* | 16 | 0.3331 | NC005942 |
|  | *Argyroneta aquatica* | 16 | 0.3470 | NC026863 |
|  | *Neoscona theisi* | 16 | 0.3235 | NC026290 |
|  | *Ornithoctonus huwena* | 15 | 0.3453 | NC005925 |
|  | *Pholcus* sp. | 13 | 0.3913 | KJ782458 |
|  | *Hypochilus thorelli* | 13 | 0.3556 | EU523753 |
|  | *Pholcus phalangioides* | 13 | 0.3863 | KJ782458 |
|  | *Liphistius erawan* | 0 | 0.2586 | NC020323 |
| Scorpiones | *Heterometrus longimanus* | 3 | 0.2420 | NC029207 |
|  | *Mesobuthus gibbosus* | 7 | 0.2738 | AJ716204 |
|  | *Mesobuthus martensii* | 5 | 0.2626 | NC009738 |
|  | *Buthus occitanus* | 5 | 0.2595 | EU523755 |
|  | *Centruroides limpidus* | 7 | 0.2501 | NC006896 |
|  | *Tityus serrulatus* | 5 | 0.2652 | NC027855 |
|  | *Uroctonus mordax* | 0 | 0.2206 | NC010782 |
|  | *Vaejovis smithi* | 0 | 0.2122 | NC035567 |
| Sarcoptiformes | *Paraleius leontonychus* | 21 | 0.3420 | LT984407 |
|  | *Oribatula tibialis* | 18 | 0.3370 | MN622223 |
|  | *Zygoribatula sp.* | 18 | 0.3399 | MN622179 |
|  | *Bipassalozetes perforatus* | 16 | 0.3491 | MN622229 |
|  | *Hermanniella granulata* | 16 | 0.3350 | MN622192 |
|  | *Oppia africana* | 16 | 0.3465 | MN622234 |
|  | *Xenillus tegeocranus* | 22 | 0.3642 | MN622216 |
|  | *Ceratoppia bipilis* | 19 | 0.3430 | MN622161 |
|  | *Lohmannia hungarorum* | 18 | 0.4243 | MN622182 |
|  | *Epilohmannia* sp. | 18 | 0.3967 | MN622165 |
|  | *Steganacarus magnus* | 20 | 0.4273 | NC011574 |
|  | *Histiostoma blomquisti* | 26 | 0.4296 | NC031377 |
|  | *Dermatophagoides farinae* | 24 | 0.4128 | NC013184 |
|  | *Sarcoptes scabiei* | 24 | 0.4459 | AP017940 |
|  | *Humerobates rostrolamellatus* | 16 | 0.3269 | MN622230 |
|  | *Acarus farris* | 24 | 0.4093 | MZ425442 |
|  | *Rhizoglyphus robini* | 24 | 0.4078 | NC038058 |
| Trombidiformes | *Sperchon plumifer* | 24 | 0.3441 | NC039813 |
|  | *Panonychus ulmi* | 31 | 0.6017 | NC012571 |
|  | *Amphitetranychus viennensis* | 31 | 0.5766 | NC032381 |
|  | *Tetranychus urticae* | 31 | 0.5931 | NC024677 |
|  | *Oligonychus shinkajii* | 31 | 0.5843 | MZ425445 |
|  | *Tetranychus malaysiensis* | 31 | 0.5839 | NC024678 |
|  | *Demodex folliculorum* | 15 | 0.4736 | NC026102 |
|  | *Trombiculidae* sp. | 23 | 0.4061 | MN622164 |
|  | *Leptotrombidium deliense* | 28 | 0.4111 | NC007600 |
|  | *Walchia hayashii* | 25 | 0.3934 | NC010595 |
|  | *Mahunkania secunda* | 29 | 0.4192 | MZ425440 |
| Eriophyoidea | *Cosella* sp. | 18 | 0.5534 | GZ75 |
|  | *Cosella viburniae.* | 19 | 0.5315 | GD139 |
|  | *Abacarus* sp. | 15 | 0.5049 | GD136 |
|  | *Aculops sinensis* | 18 | 0.5057 | HEN18 |
|  | *Neocalepitrimerus rosa* | 15 | 0.5054 | GX37 |
|  | *Surapoda tianlinensis* | 17 | 0.5230 | Z302 |
|  | Diptilomiopinae sp. | 20 | 0.4753 | GZ126 |
|  | *Trimeroptes luanchuanensis* | 22 | 0.4642 | Z322 |
|  | *Epitrimerus sabinae* | 19 | 0.4724 | GZ121 |
|  | *Epitrimerus* sp. | 15 | 0.4748 | S153 |
|  | *Eriophyes armandis* | 13 | 0.4725 | Z303 |
|  | *Rhyncaphytoptus redwayensis* | 15 | 0.4819 | CA28 |
|  | *Tegolophus tremae* | 18 | 0.4796 | Z368 |
|  | *Scolotosus* sp. | 18 | 0.5072 | GZ100 |
|  | *Aceria aloinis* | 18 | 0.4623 | BJ2 |
|  | *Nalepella abiesis* | 18 | 0.4841 | Z275 |
|  | *Setoptus koraiensis* | 18 | 0.4759 | SD23 |
|  | *Boczekella fabris* | 18 | 0.4650 | HLJ101 |
|  | *Trisetacus ehmanni* | 16 | 0.4794 | Z341 |
| Pseudoscorpionida | *Paratemnoides elongatus* | 12 | 0.3900 | NC017752 |
|  | *Pseudogarypus banksi* | 18 | 0.4125 | JQ040544 |
| Pycnogonida | *Achelia bituberculata* | 3 | 0.2818 | AY457170 |
|  | *Tanystylum orbiculare* | 3 | 0.2951 | GU370074 |
|  | *Ammothea carolinensis* | 3 | 0.2763 | GU065293 |
|  | *Nymphon gracile* | 20 | 0.3536 | DQ666063 |
