# Supplementary material for: Phylogenomics resolves the higher-level phylogeny of herbivorous eriophyoid mites (Acariformes: Eriophyoidea)
Source: BMC Biol. 2024 Mar 22;22:70. doi: 10.1186/s12915-024-01870-9 (PMC10960459; doi:10.1186/s12915-024-01870-9)

Additional file 2: Fig. S1. The 54 mitochondrial gene arrangement patterns (Pattern 1-Pattern 54) from 153 eriophyoid mite species in this study. Genes underlined have opposite transcription orientation to those not underlined. Translocated or inverted genes are colour-coded (blue: inversion and translocation; green: translocation; orange: inversion). Abbreviations of protein-coding genes are *atp6* and *atp8* for ATP synthase subunits 6 and 8; *cox1–3* for cytochrome oxidase subunits 1–3; *cob* for cytochrome b; *nad1–6* and *nad4L* for NADH dehydrogenase subunits 1–6 and 4L; *rrnL* and *rrnS* for large and small rRNA subunits; tRNA genes are indicated by the single-letter IUPAC-IUB abbreviations for their corresponding amino acids. The shared rearranged mt gene clusters of each sample were denoted by underneath lines in different colors as the same as Figure 1.

Hypothetical ancestor of arthropods

Phytoptidae: Pattern 1 (*Boczekella fabris*_HLJ101, *Nalepella abiesis*_Z275, *Setoptus koraiensis*_SD23)

Phytoptidae: Pattern 2 (*Trisetacus ehmanni*_Z341)

Phytoptidae: Pattern 3 (*Fragariocoptes setiger*)

Nothopodinae (*Surapoda* spp.): Pattern 4 (*Surapoda* sp._GD126, *Surapoda tianlinensis*_Z302)

Nothopodinae (*Cosella* spp.): Pattern 5 (*Cosella* sp._GZ75)

Nothopodinae (*Cosella* spp.): Pattern 6 (*Cosella* sp._FJ16)

Nothopodinae (*Cosella* spp.): Pattern 7 (*Cosella viburniae*_GD139)

Diptilomiopinae: Pattern 8 (Apodiptacus sp._E11)

Diptilomiopinae: Pattern 9 (*Trimeroptes luanchuanensis*_Z322)

Diptilomiopinae: Pattern 10 (Diptilomiopinae sp._GZ38, *Apodiptacus toxicodendri*_FJ25)

Diptilomiopinae: Pattern 11 (*Acarhis* sp._Z277)

Diptilomiopinae: Pattern 12 (Diptilomiopinae sp._GZ126)

Diptilomiopinae: Pattern 13 (*Diptilomiopus* sp._LN6)

Diptilomiopinae: Pattern 14 (*Diptacus* sp._CA46)

Diptilomiopinae: Pattern 15 (*Diptilomiopus* sp._Z388)

Diptilomiopinae: Pattern 16 (*Diptacus brevichaetus*_Z365)

Diptilomiopinae: Pattern 17 (*Apodiptacus castaneae*_Z377)

RCP: Pattern 18 (*Rhyncaphytoptus* sp._GS9, *Rhinotergum shaoguanense*_S88, *Rhyncaphytoptus redwayensis*_CA28, *Rhyncaphytoptus ulmi*_HEN3, *Rhinophytoptus* c.f. *sericeaomeiensis*_AH42, *Quadracus cudraniae*_AH41, *Leipothrix* sp._Z372, *Epitrimerus* sp._SD25)

RCP: Pattern 19 (*Rhinophytoptus broussonetiae*_GZ98, *Rhinophytoptus broussonetiae*_Z401, *Rhinophytoptus broussonetiae*_GZ74)

RCP: Pattern 20 (*Rhyncaphytoptus* sp._GZ96)

RCP: Pattern 21 (*Rhinophytoptus* c.f. *broussonetiae*_Z292)

RCP: Pattern 22 (*Keiferella guanegouensis*_QH1)

RCP: Pattern 23 (*Phyllocoptes taishanensis*_S90, *Proiectus granularpro*_Z304, *Eriophyes armandis*_Z303)

RCP: Pattern 24 (*Neoepitrimerus platyclad*_S138-16S, *Leipothrix* c.f. *sabinae*_HEN4, *Epitrimerus* sp._S153)

RCP: Pattern 25 (*Phyllocoptes* c.f. *taishanensis*_S167)

RCP: Pattern 26 (*Phyllocoptrata platyclada*_S138-12S, *Phyllocoptrata platyclada*_G6, *Epitrimerus* c.f. *sabinae*_HEN25, *Epitrimerus* c.f. *sabinae*_S83)

RCP: Pattern 27 (*Tegolophus* sp._CA38, *Tegolophus ringsi*_CA37)

RCP: Pattern 28 (*Paracalacarus podocarpi*_S142)

RCP: Pattern 29 (*Calepitrimerus* sp._Z308)

RCP: Pattern 30 (*Litaculus* sp._Z392)

RCP: Pattern 31 (*Epitrimerus* c.f. *sabinae*_Z343)

RCP: Pattern 32 (*Leipothrix* sp._S83)

RCP: Pattern 33 (*Epitrimerus* c.f. *sabinae*_Z300, *Epitrimerus sabinae*_GZ121, *Epitrimerus* c.f. *sabinae*_Z344)

RCP: Pattern 34 (*Fujianacarus wisterianis*_CQ1, *Fujianacarus wisterianis*_HN237, *Fujianacarus wisterianis*_Z399)

RCP: Pattern 35 (*Tegolophus* c.f. *miscanthus*_Z371)

RCP: Pattern 36 (*Grandiscapitis camelliae*_YN453)

RCP: Pattern 37 (*Abacarus* c.f. *oplismeni*_GD121)

RCP: Pattern 38 (*Abacarus* sp._GD136)

RCP: Pattern 39 (*Aculops* c.f. *taihangensis*_SD6)

RCP: Pattern 40 (*Tegolophus suffruticosae*_Z382)

RCP: Pattern 41 (*Tegolophus* c.f. *suffruticosae*_LN14)

RCP: Pattern 42 (*Cecidophyes* sp._Z394)

RCP: Pattern 43 (*Glyptacus* sp._CA39W)

RCP: Pattern 44 (*Neocalepitrimerus rosa*_GX37, *Prominens photiniae*_FJ26)

RCP: Pattern 45 (*Cosetacus* sp._GD115)

ATA: Pattern 46 (*Aculus huzhongsalixus*_HEN37, *Aculops taihangensis*_Z289,*Tetra louguantais*_AH43, *Shevtchenkella miscanthis*_KK11, *Aculus ichnocarpi*_GZ134, *Aculops lobuliferus*_CA42, *Tetra cuihuae*_QH6, *Aculus coggygriae*_HEN32, *Aculus populi*_HEN13, *Vittacus cannabus*_HEN44, *Scolotosus* sp._GX54, *Scolotosus* sp._GZ100,*Tegolophus hunanensis*_AH39,*Tegolophus* sp._HK8, *Aculus ligustri*_HEN33, *Tegolophus hunanensis*_S152, *Tegolophus hunanensis*_HEN17, *Tegolophus hunanensis*_HEN41, *Aceria pseudoligustri*_FJ31, *Tetra suspensae*_SD22, *Tetra pyriana*_HLJ98, *Aculops sinensis*_HEN18, *Aculops* sp._CA13, *Tetra lucidi*_HEN8, *Tegolophus oblatus*_QH7, *Aculus* sp._HEN16, *Abacarus* sp._GD111, *Abacarus* sp._HEN47, *Abacarus* sp._HEN45, *Abacarus* c.f. *oplismeni*_GZ33, *Tegolophus miscanthus*_Z336, *Aculus schlechtendali*_CA18, *Aceria aloinis*_BJ2, *Abacarus* sp._CA9, *Aculus tetanothrix*_QH2, *Proiectus thunbergis*_HEN28, *Tetra* sp._Z284, *Rhyncaphytoptus longipalpis*_JX3, *Rhyncaphytoptus longipalpis*_JX2, *Aculus bambusae*_SD15, *Tetraspinus* sp._HEN26, *Tegolophus ulmi*_CQ4, *Abacarus bambusae*_GD118, *Abacarus* sp._HK3, *Abacarus bambusae*_GD119, *Tegonotus celtis*_YN448, *Tegolophus tremae*_Z368, *Tegolophus lespedezae*_HEN12, *Phyllocoptruta* sp._CA6, *Tetra qingyuanensis*_Z333, *Aculus populi*_HEN23, *Aculus* c.f. *populi*_HEN13_MT2, *Epitrimerus* sp._Z338, *Aculops* sp._Z332, *Tetra* sp._CA43, *Vasates* sp._Z327, *Tetra* sp._Z400, *Aculus* sp._Z316, *Aculops* sp._CA47, *Aculops* sp._QH8, *Calepitrimerus bungeanus*_HLJ97, *Cereusacarus* sp._Z361, *Aculus* c.f. *schlechtendali*_SD19, *Abacarus* c.f. *sacchari*_Z318, *Aceria* sp._SD20 *Tetra* sp._SD27, *Tegolophus fargesiae*_E1, *Tegolophus alangii*_Z296, *Aculusichnocarpi*_GZ124)

ATA: Pattern 47 (*Abacarus* c.f. *oplismeni*_GD129, *Abacarus sacchari*_GD130, *Dipentamerus litseae*_GD140)

ATA: Pattern 48 (*Tegolophus celtis*_S136)

ATA: Pattern 49 (*Acaphyllisa fagi*_CA35)

ATA: Pattern 50 (*Paraepitrimerus* sp._H93, *Tetra* sp._GZ35, *Tetra lycopersici*_FQ)

ATA: Pattern 51 (*Epitrimerus* c.f. *spirae*_KK1)

"*Calepitrimerus s.l.*" : Pattern 52 (*Epitrimerus pyri*_AH40)

"*Calepitrimerus s.l.*" : Pattern 53 (*Calepitrimerus anomalus*_HLJ99)

"*Calepitrimerus s.l.*" : Pattern 54 (*Calepitrimerus flexuosus*_Z342, *Calepitrimerus* sp._CA2)

Additional file 2: Fig. S2. Mitochondrial gene arrangements of representative samples in the Eriophyoidea. Two samples were selected as representatives of each clade. Shared rearranged mt gene clusters of each sample were denoted by underneath lines in different colors. Underlined genes are encoded in the N-strand. Translocated or inverted genes are colour-coded (blue: inversion and translocation; green: translocation; orange: inversion). Abbreviations of protein-coding genes are *atp6* and *atp8* for ATP synthase subunits 6 and 8; *cox1–3* for cytochrome oxidase subunits 1–3; *cob* for cytochrome b; *nad1–6* and *nad4L* for NADH dehydrogenase subunits 1–6 and 4L; *rrnL* and *rrnS* for large and small rRNA subunits; tRNA genes are indicated by the single-letter IUPAC-IUB abbreviations for their corresponding amino acids.

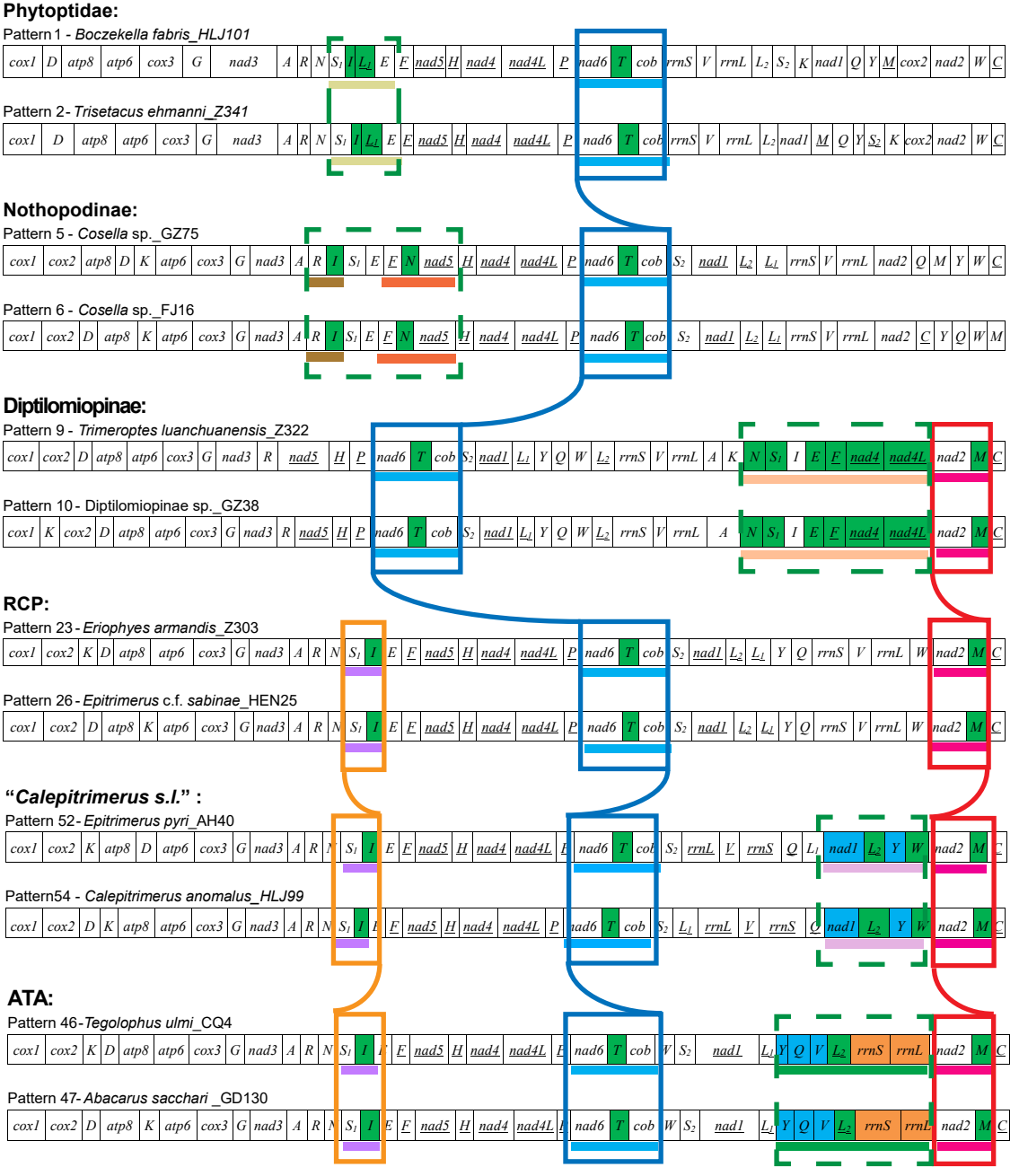

Additional file 2: Fig. S3. The phylogenetic tree inferred from mitochondrial genome nucleotide sequences using maximum likelihood method with partition by gene and GTRGAMMAI model.

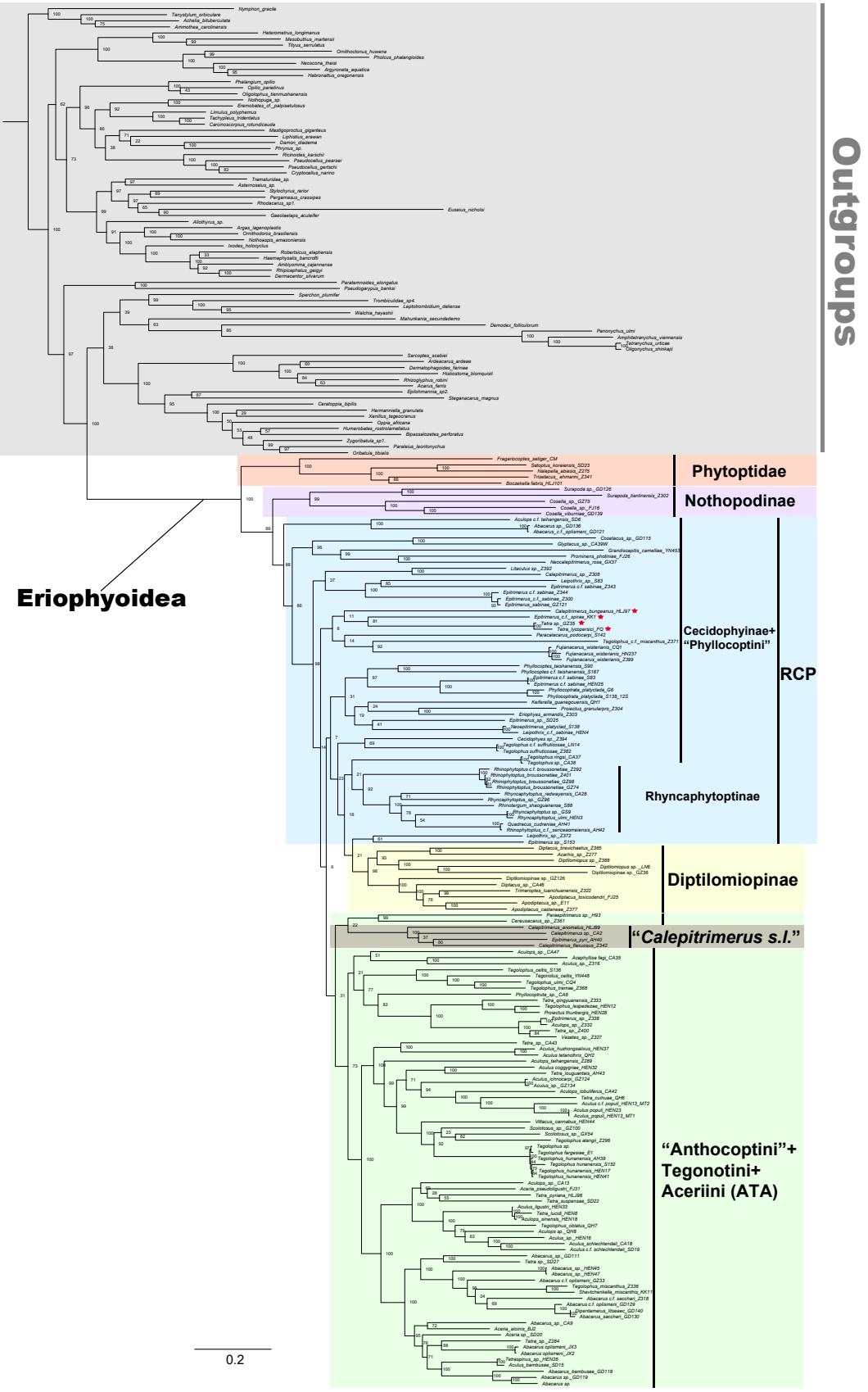

Additional file 2: Fig. S4. The phylogenetic tree inferred from mitochondrial genome nucleotide sequences (without the 3rd codon positions of PCGs) using maximum likelihood method with partition by gene and GTRGAMMAI model.

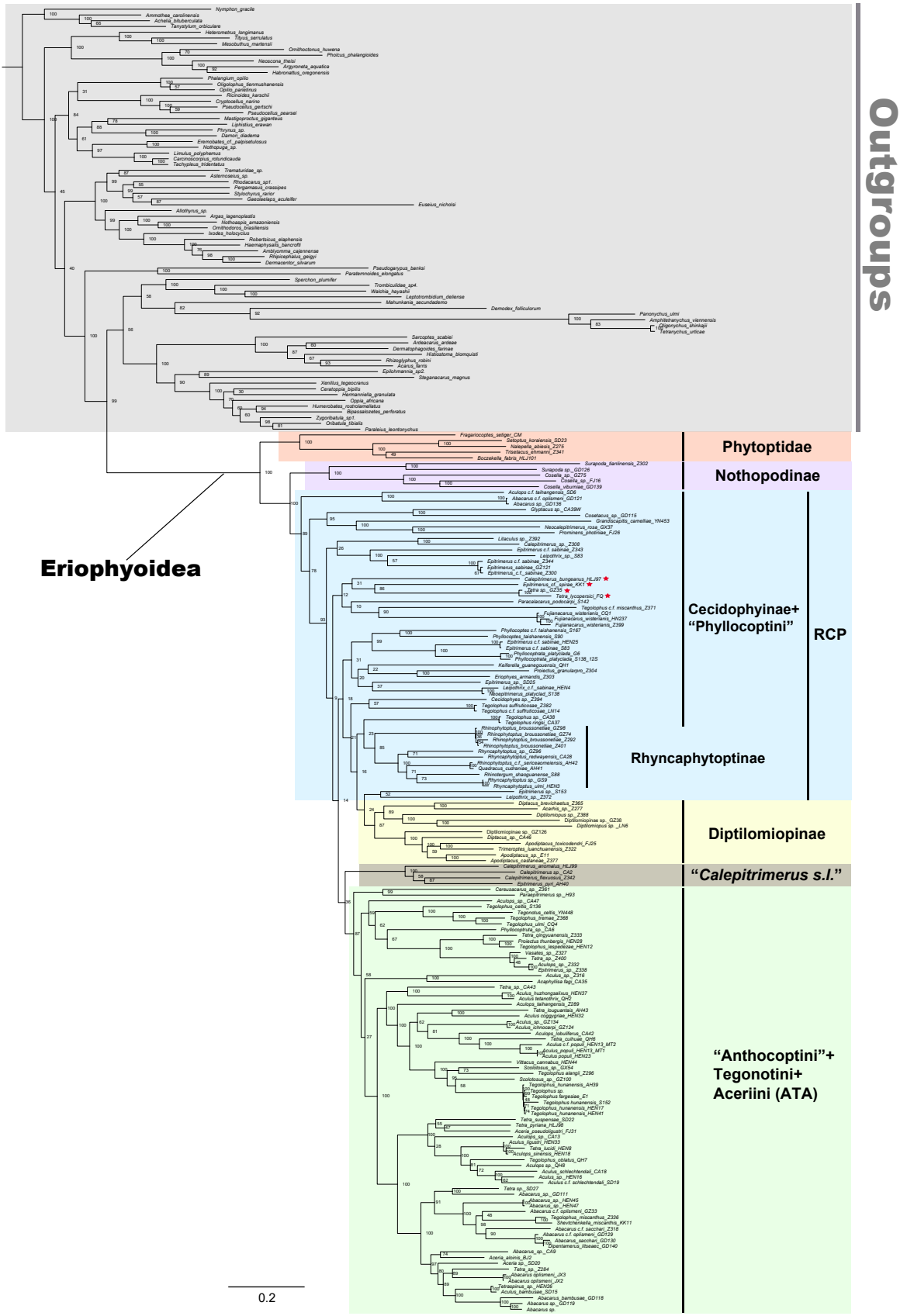

Additional file 2: Fig. S5. The phylogenetic tree inferred from mitochondrial genome nucleotide sequences using maximum likelihood method with partition by codon and GTRGAMMAI model.

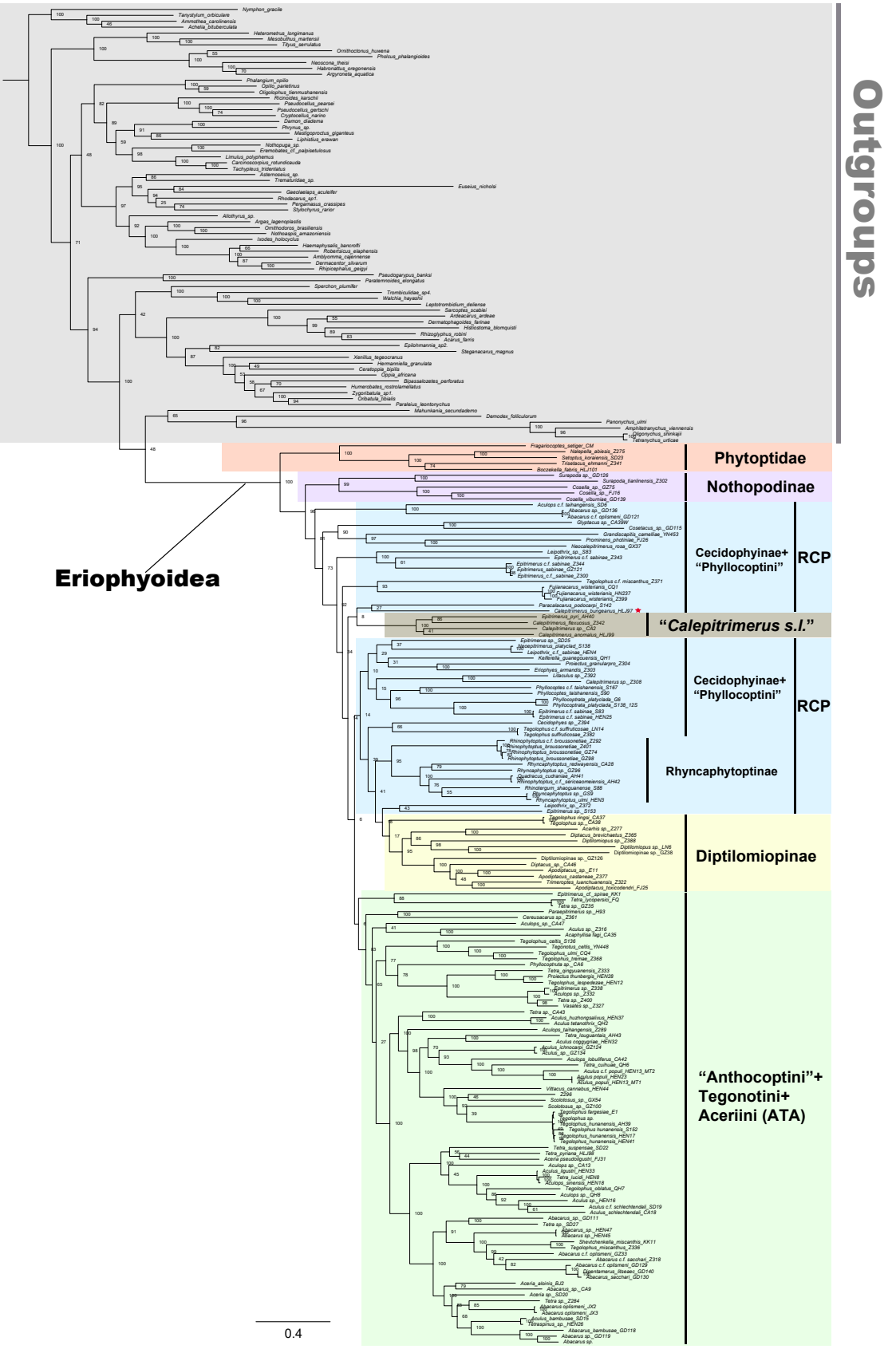

Additional file 2: Fig. S6. The phylogenetic tree inferred from mitochondrial genome nucleotide sequences (without the 3rd codon positions of PCGs) using maximum likelihood method with partition by codon and GTRGAMMAI model.

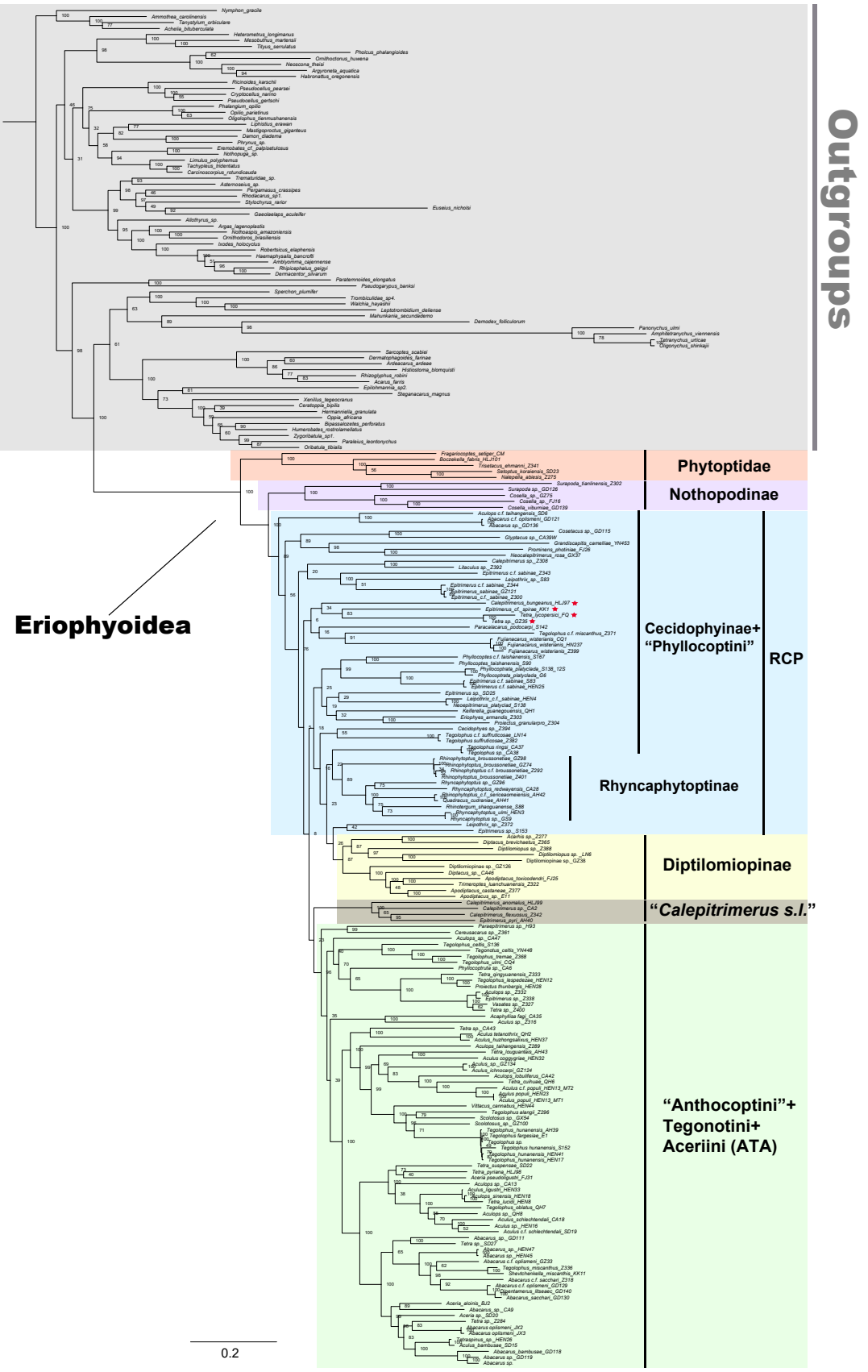

Additional file 2: Fig. S7. The phylogenetic tree inferred from mitochondrial genome nucleotide sequences using maximum likelihood method with partition by PartitionFinder and GTRGAMMAI model.

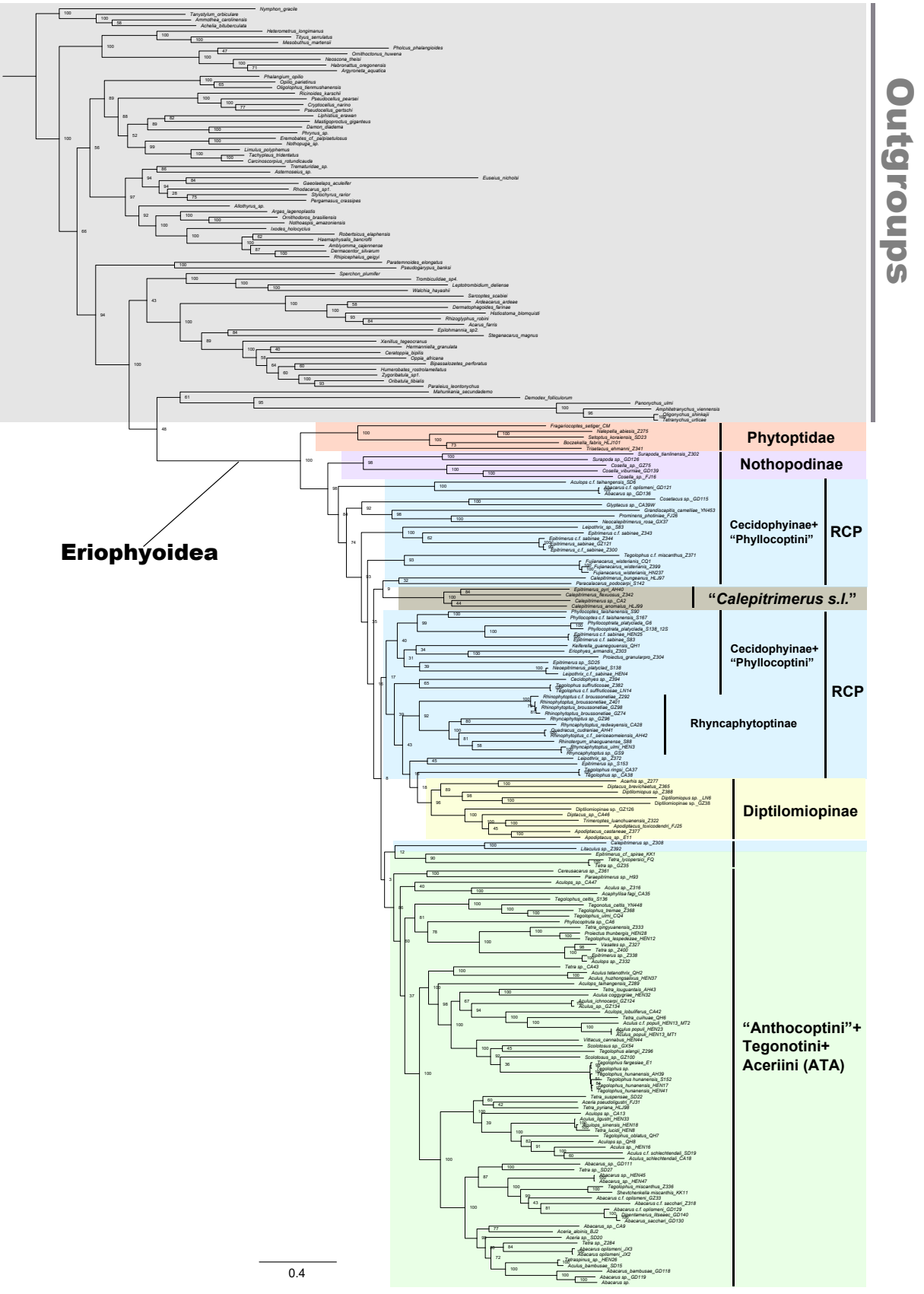

Additional file 2: Fig. S8. The phylogenetic tree inferred from mitochondrial genome amino acid sequences using maximum likelihood method with partition by gene and GAMMA model.

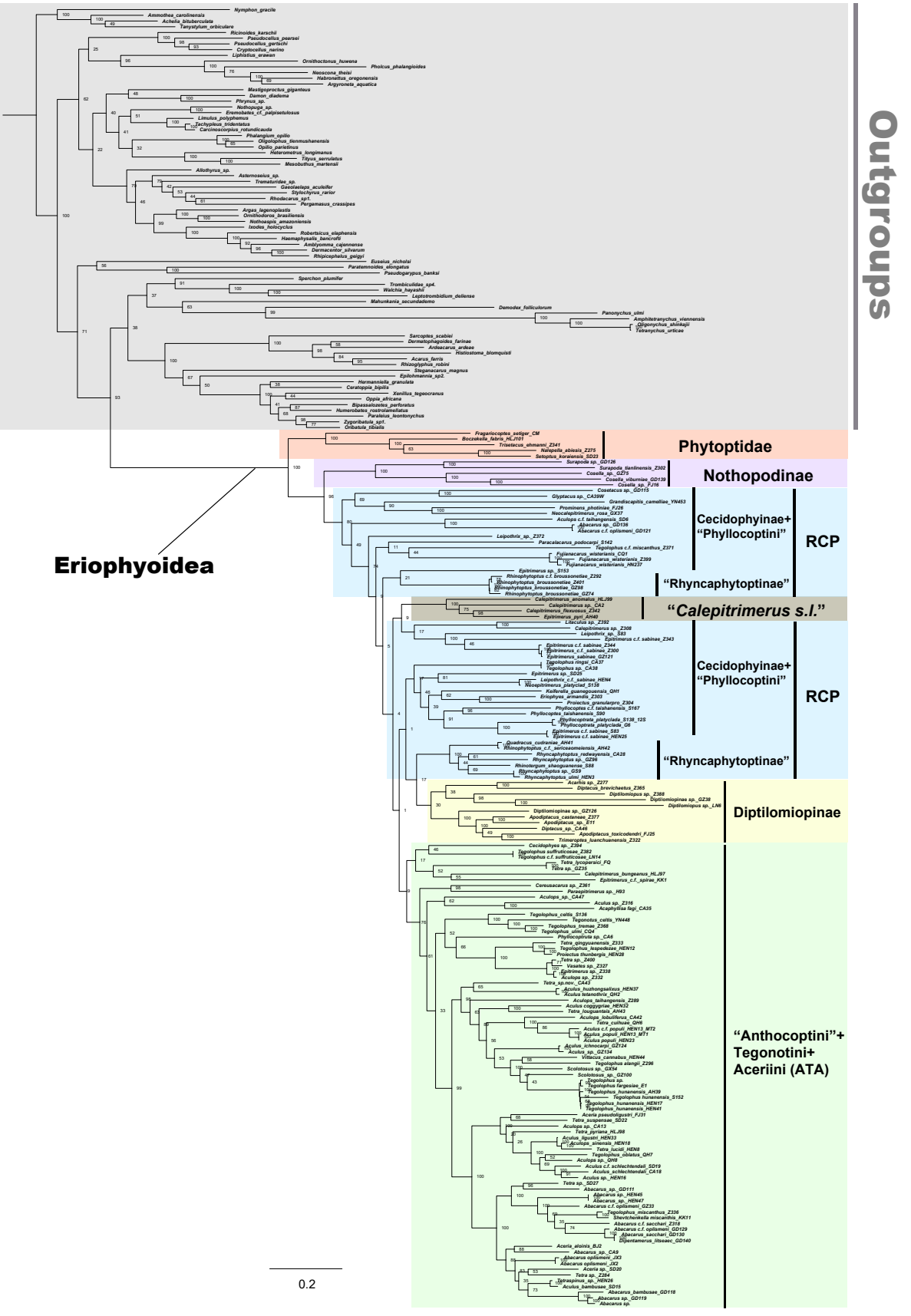

Additional file 2: Fig. S9. The phylogenetic tree inferred from mitochondrial genome amino acid sequences using maximum likelihood method with partition by PartitionFinder and GAMMA model.

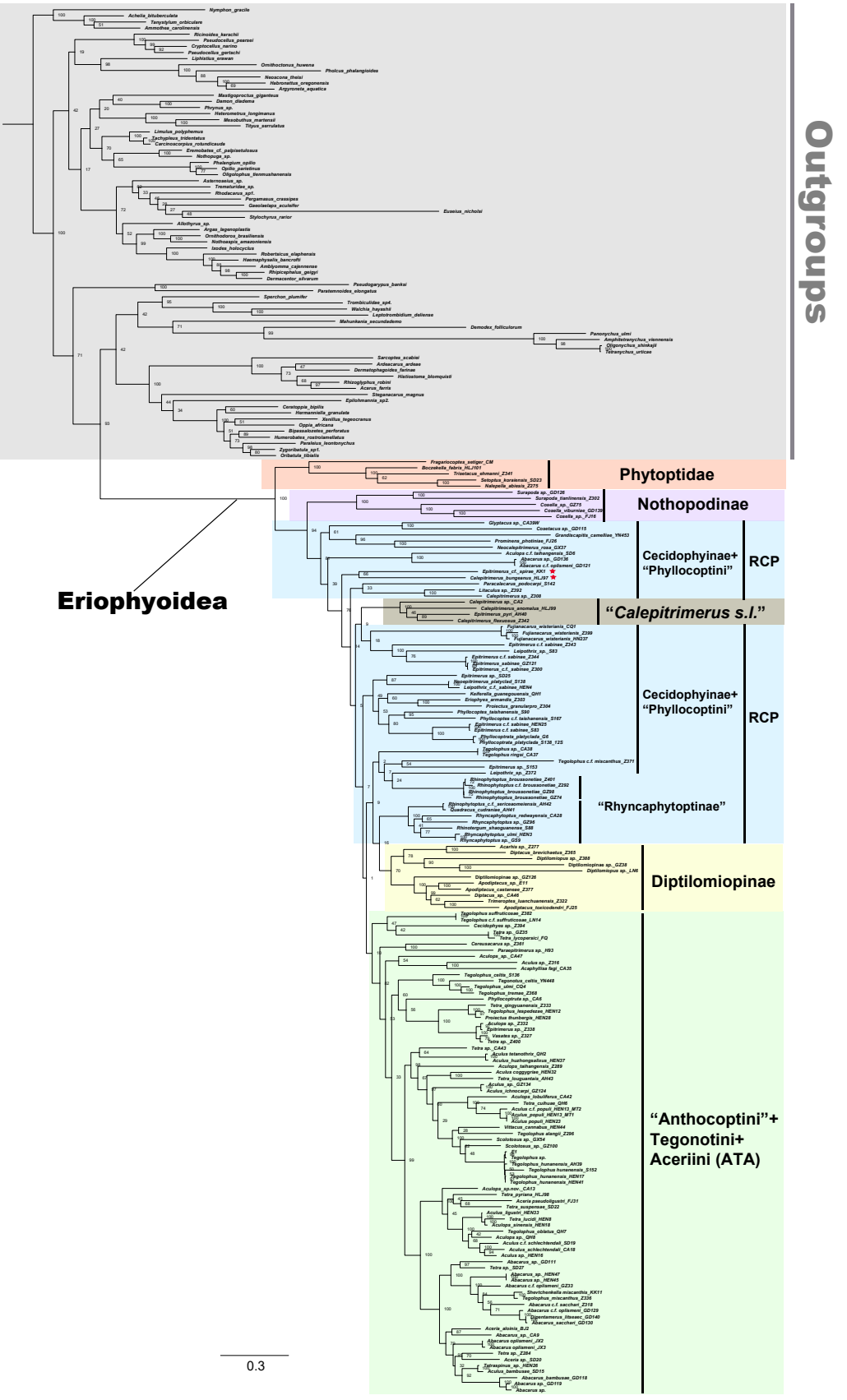

Additional file 2: Fig. S10. The phylogenetic tree inferred from mitochondrial genome nucleotide sequences using maximum likelihood method with partition by gene and GTRCAT model.

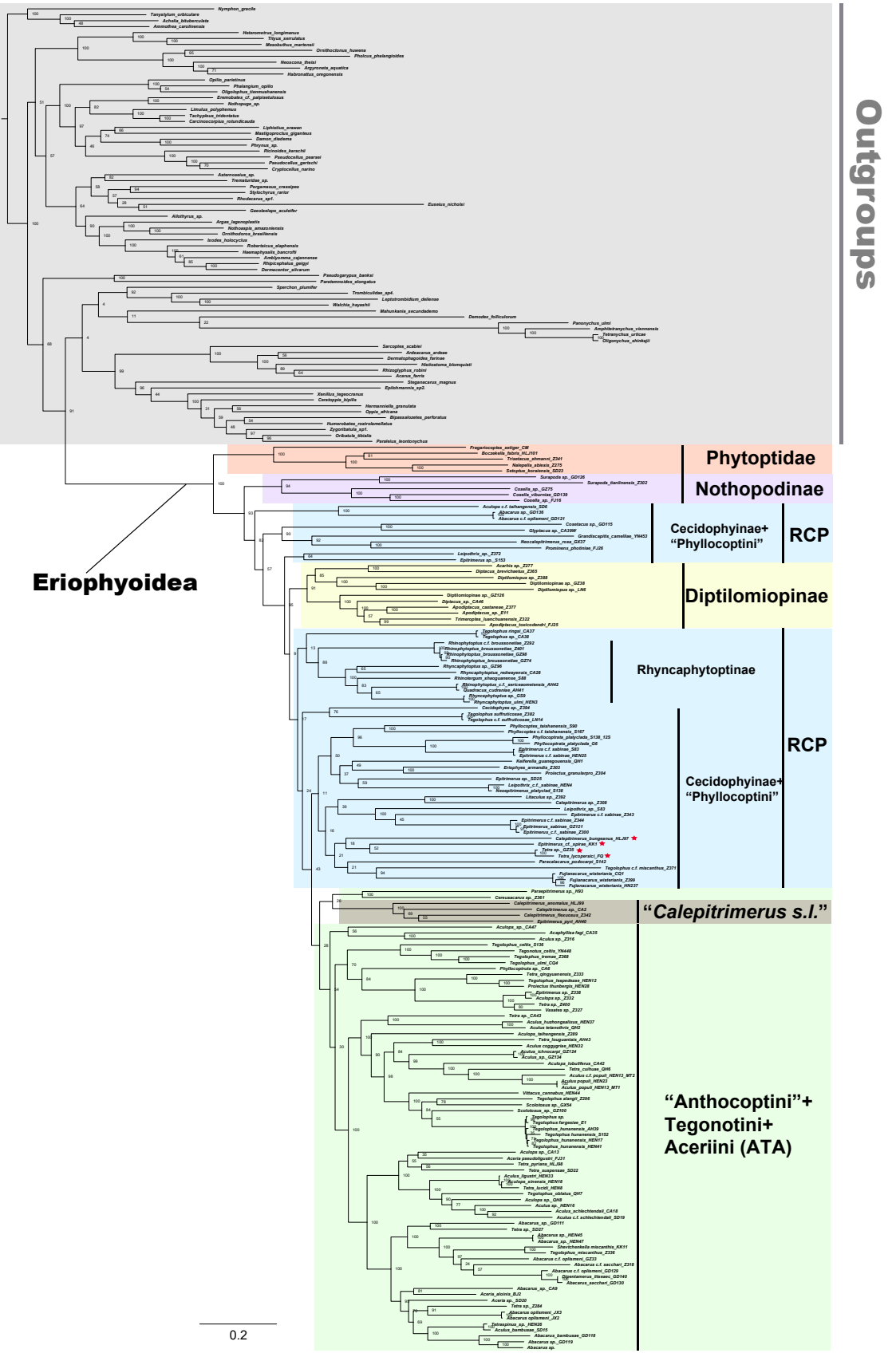

Additional file 2: Fig. S11. The phylogenetic tree inferred from mitochondrial genome nucleotide sequences (without the 3rd codon positions of PCGs) using maximum likelihood method with partition by gene and GTRCAT model.

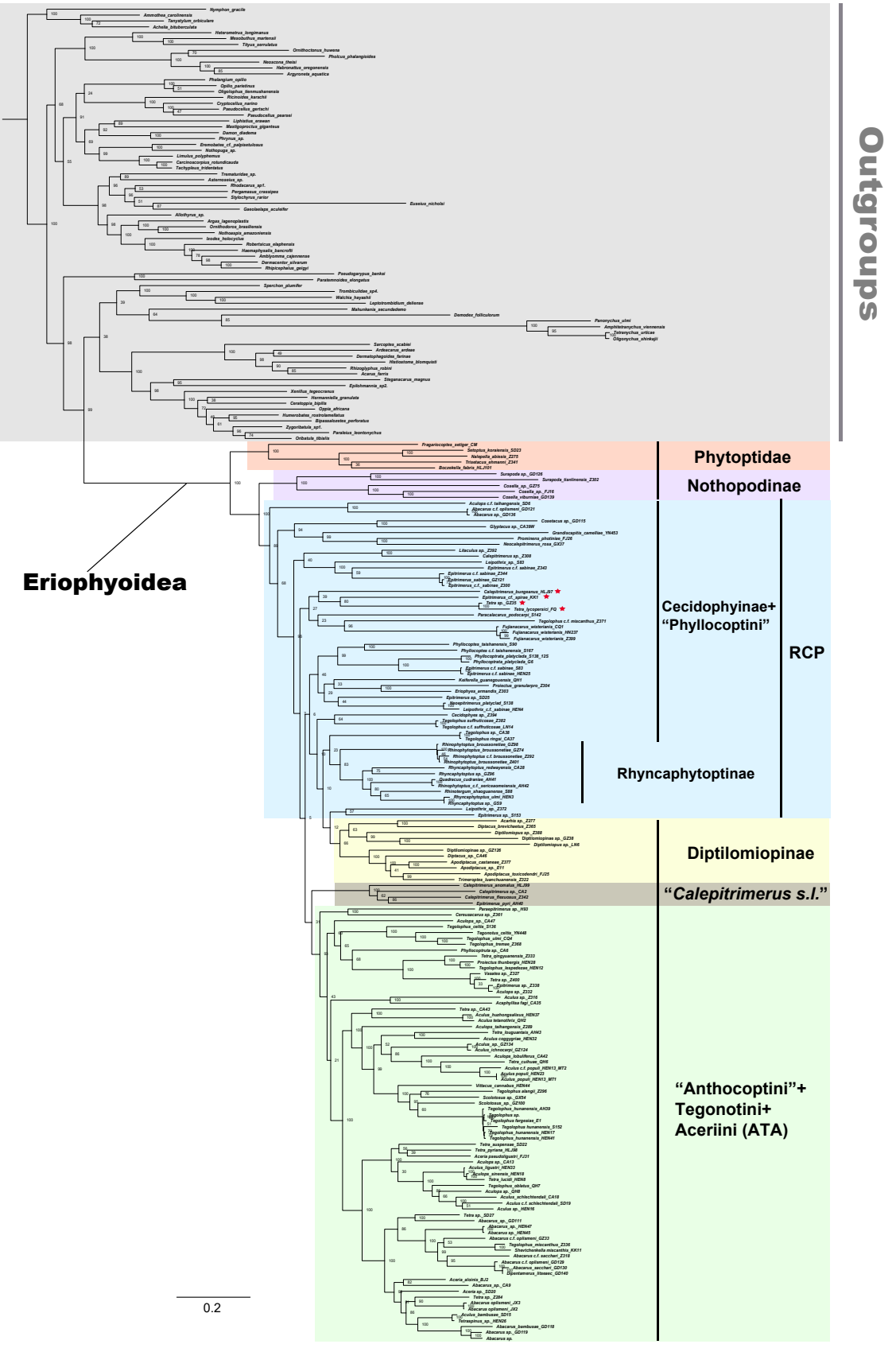

Additional file 2: Fig. S12. The phylogenetic tree inferred from mitochondrial genome nucleotide sequences using maximum likelihood method with partition by codon and GTRCAT model.

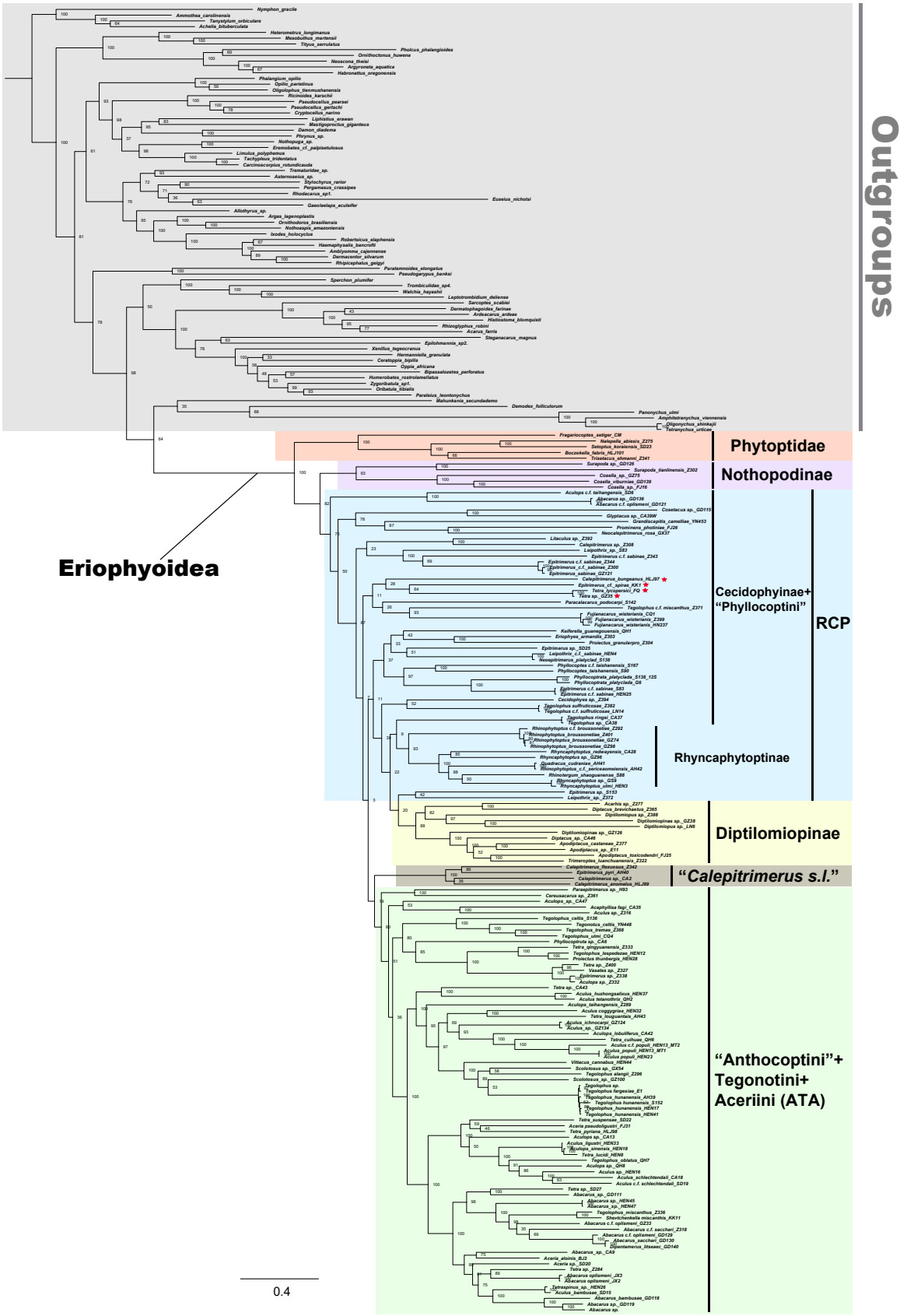

Additional file 2: Fig. S13. The phylogenetic tree inferred from mitochondrial genome nucleotide sequences (without 3rd codon positions of PCGs) using maximum likelihood method with partition by codon and GTRCAT model.

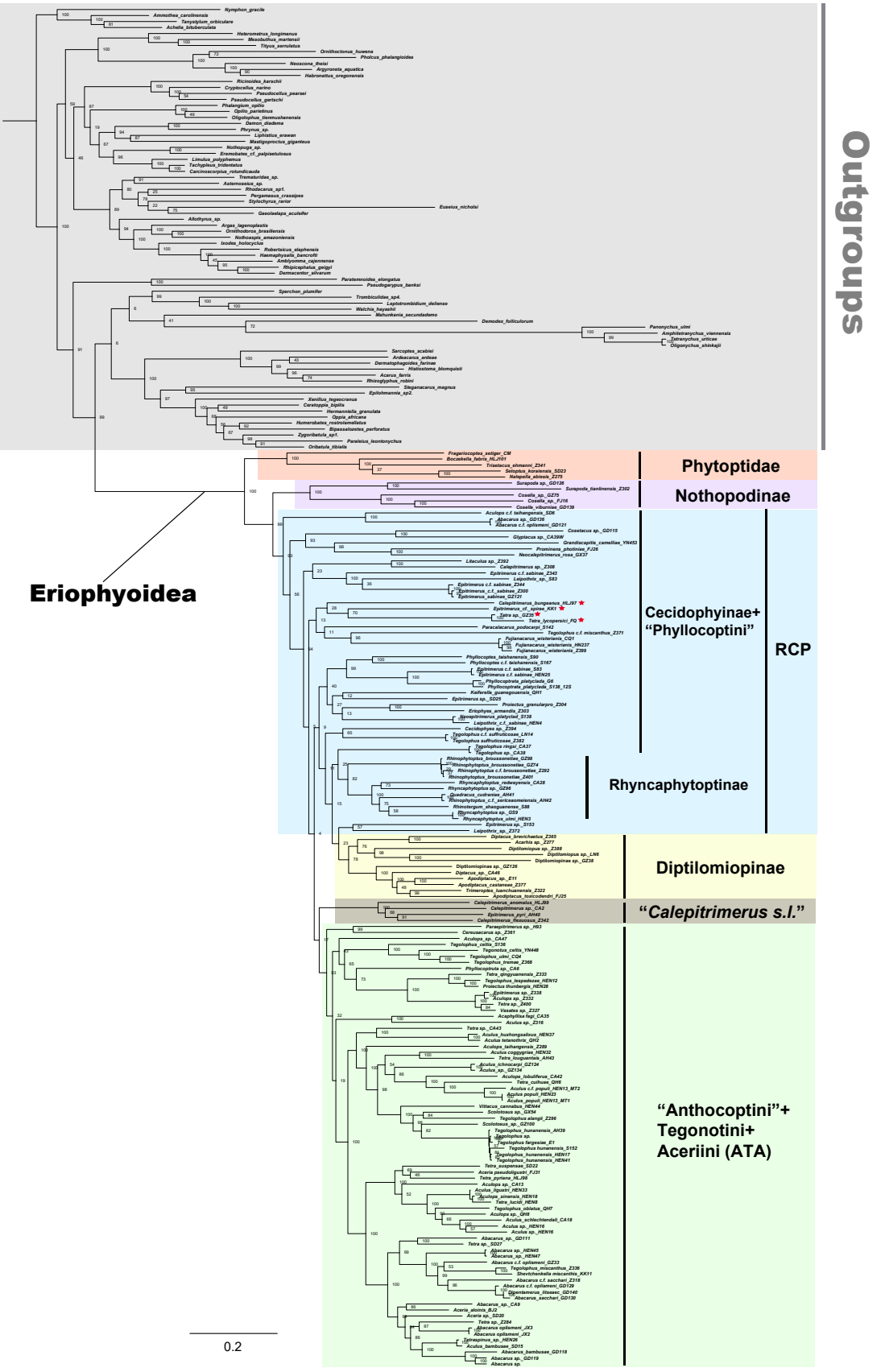

Additional file 2: Fig. S14. The phylogenetic tree inferred from mitochondrial genome nucleotide sequences using maximum likelihood method with partition by PartitionFinder and GTRCAT model.

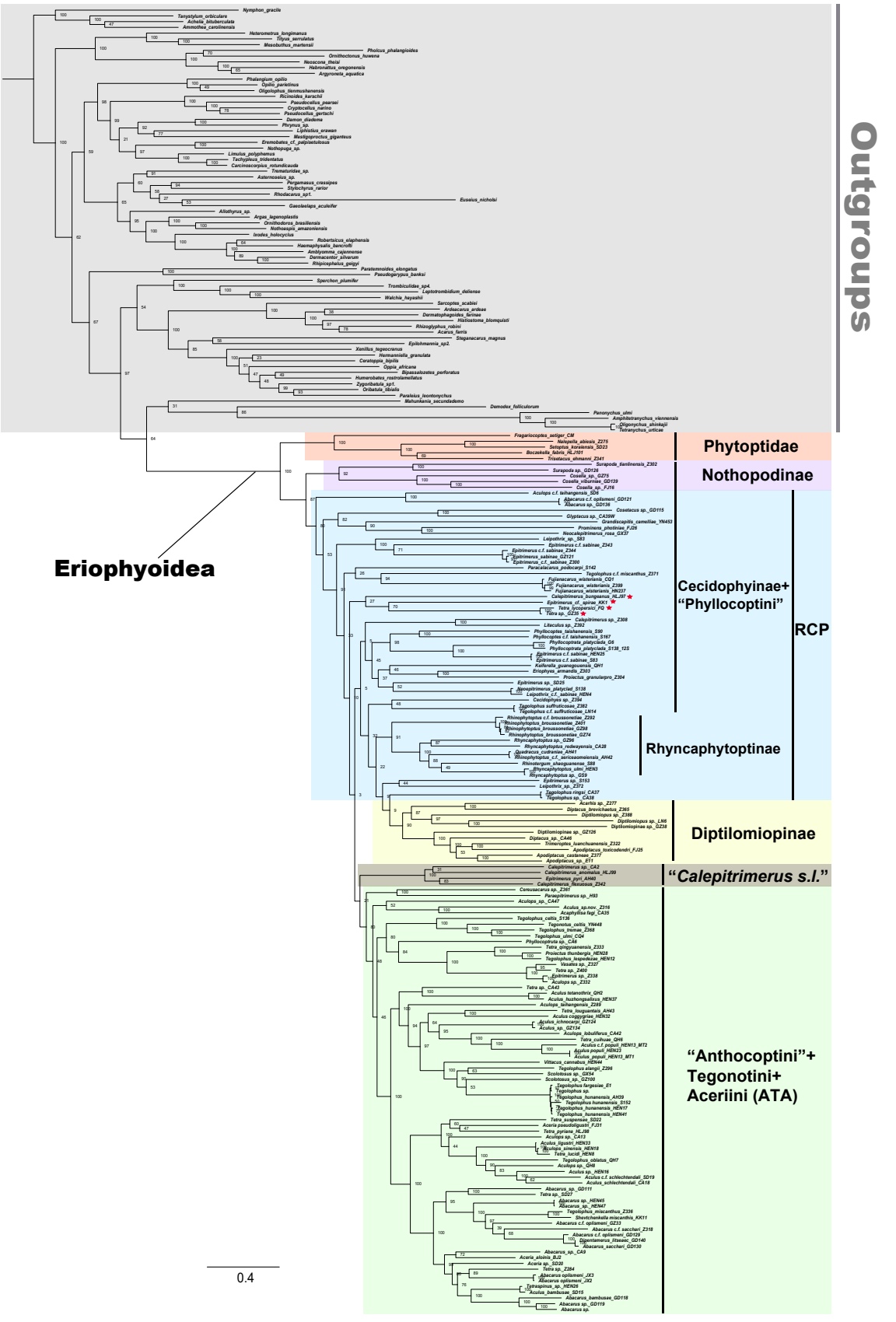

Additional file 2: Fig. S15. The phylogenetic tree inferred from mitochondrial genome amino acid sequences using maximum likelihood method with partition by gene and GTRCAT model.

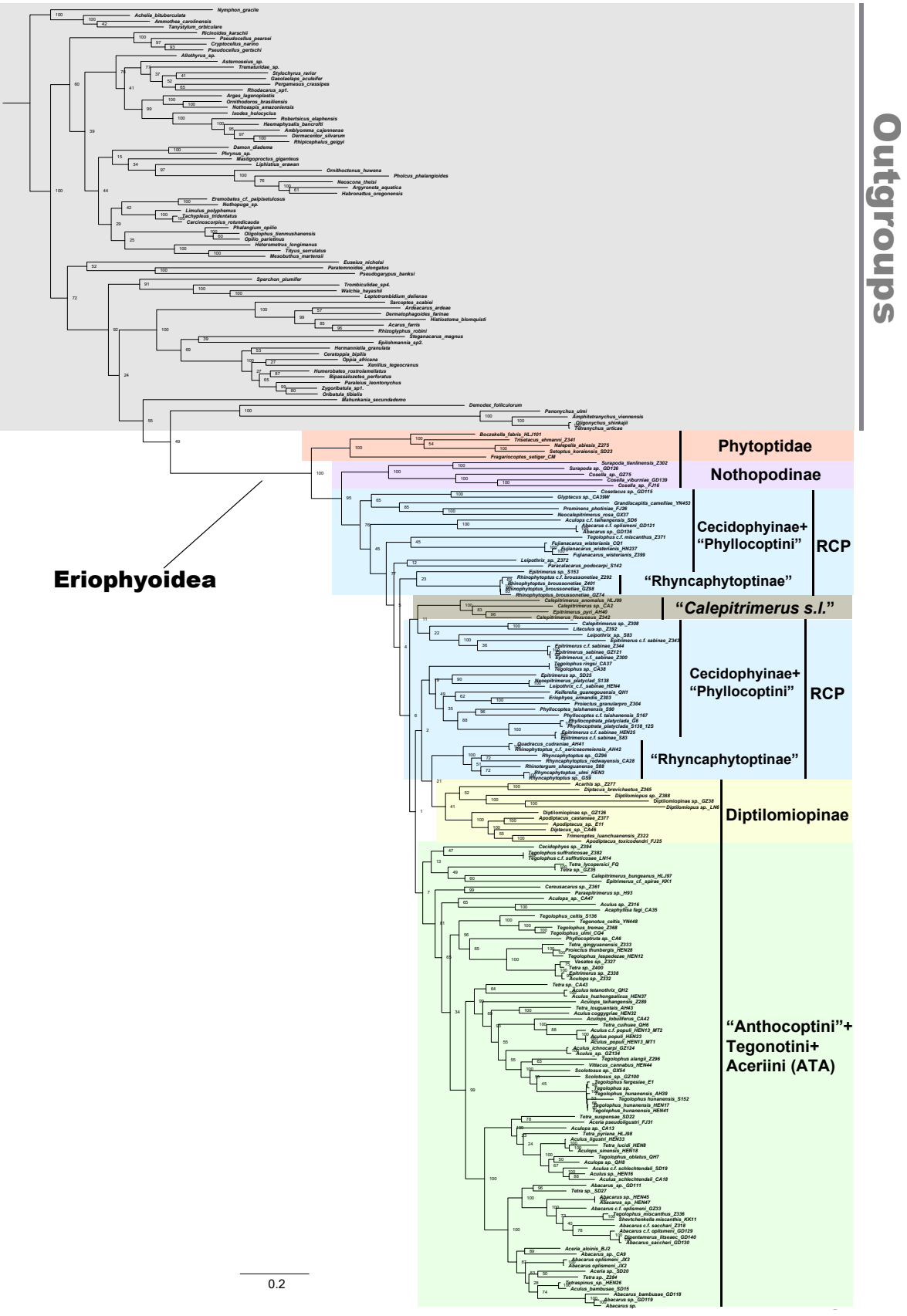

Additional file 2: Fig. S16. The phylogenetic tree inferred from mitochondrial genome amino acid sequences using maximum likelihood method with partition by PartitionFinder and GTRCAT model.

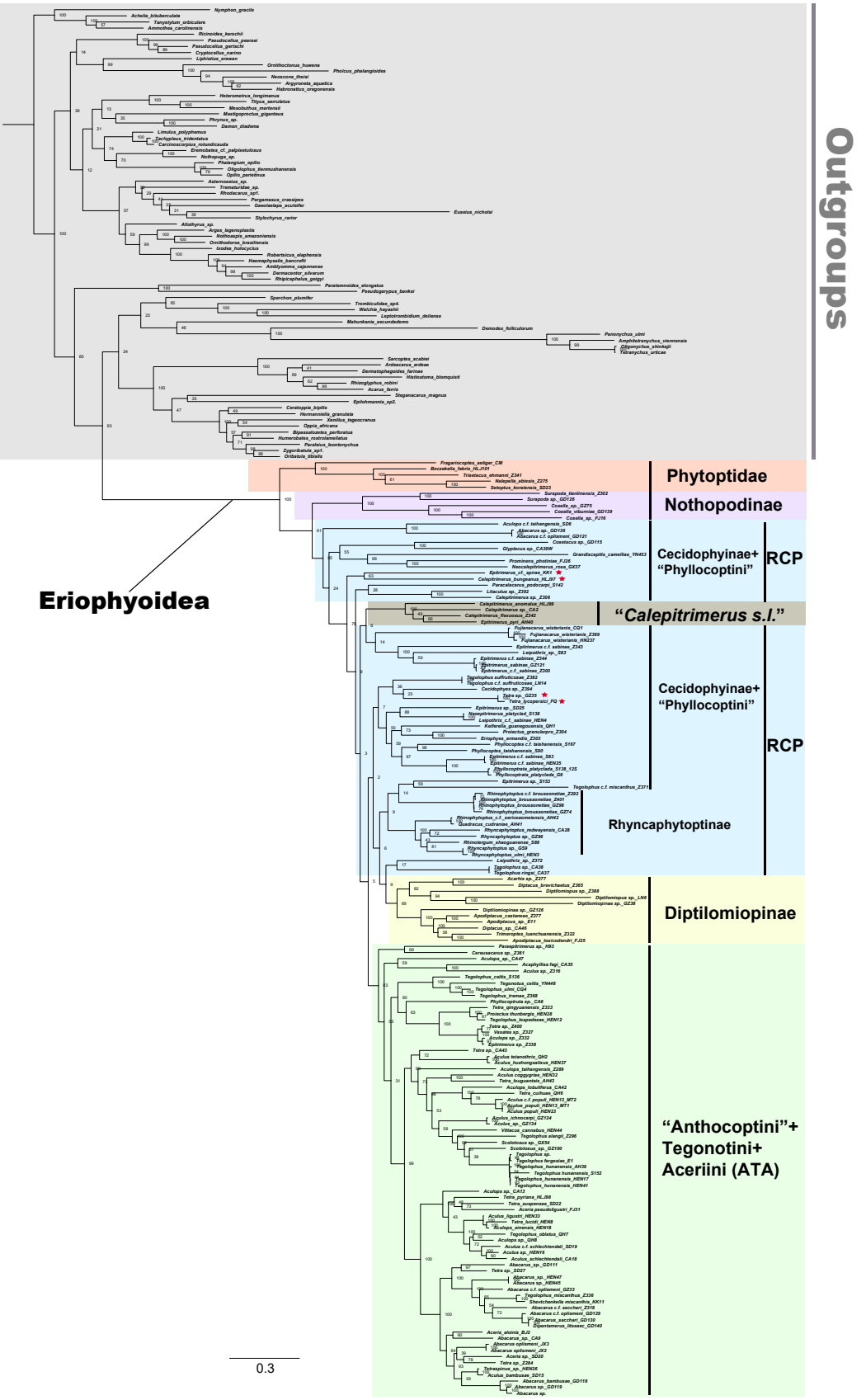

Additional file 2: Fig. S17. The phylogenetic tree inferred from mitochondrial genome nucleotide sequences (without 3rd codon positions of PCGs) using Bayesian method and partition by gene.

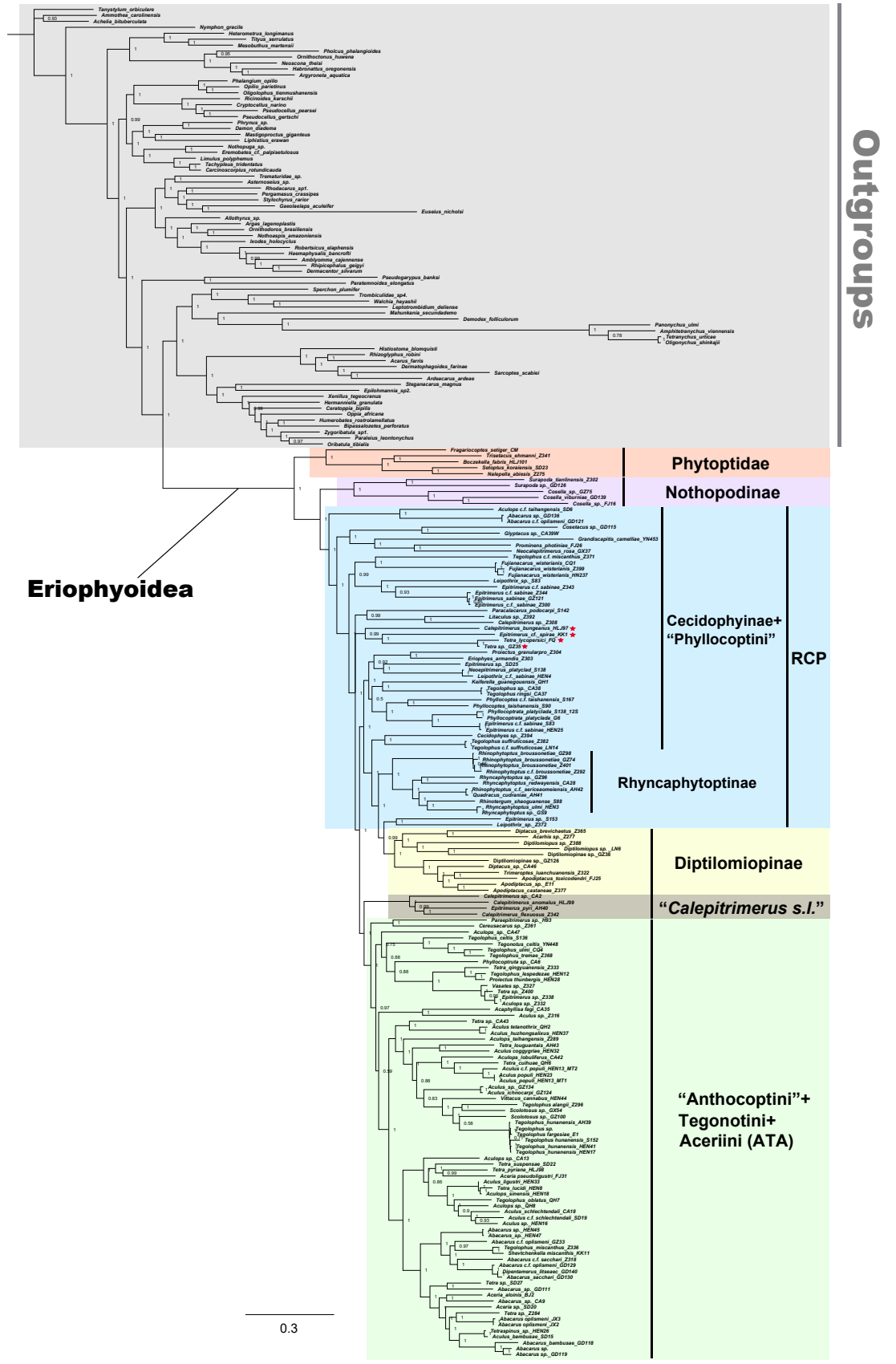

Additional file 2: Fig. S18. The phylogenetic tree inferred from mitochondrial genome nucleotide sequences using Bayesian method and partition by codon.

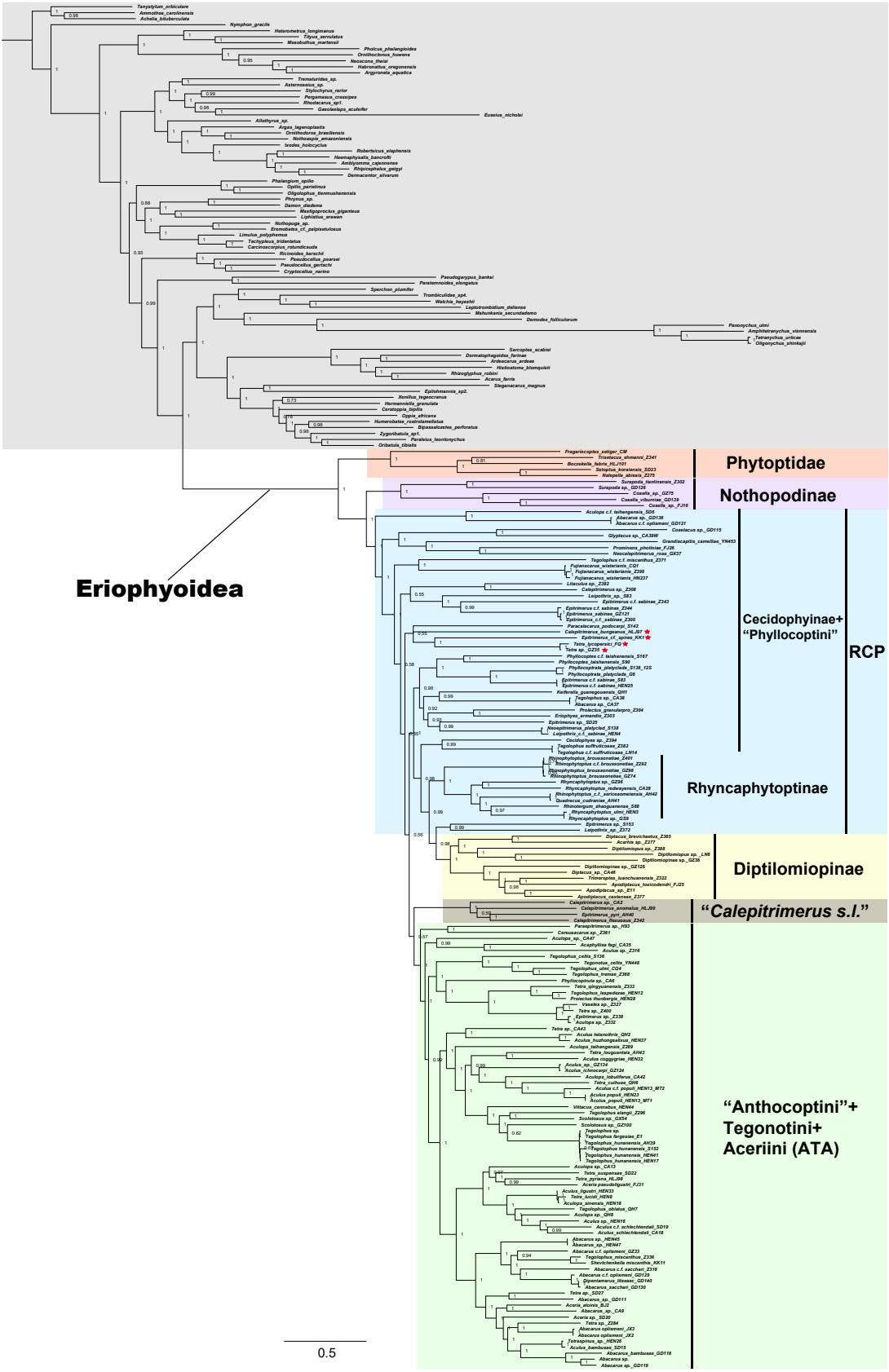

Additional file 2: Fig. S19. The phylogenetic tree inferred from mitochondrial genome nucleotide sequences (without 3rd codon positions of PCGs) using Bayesian method and partition by codon.

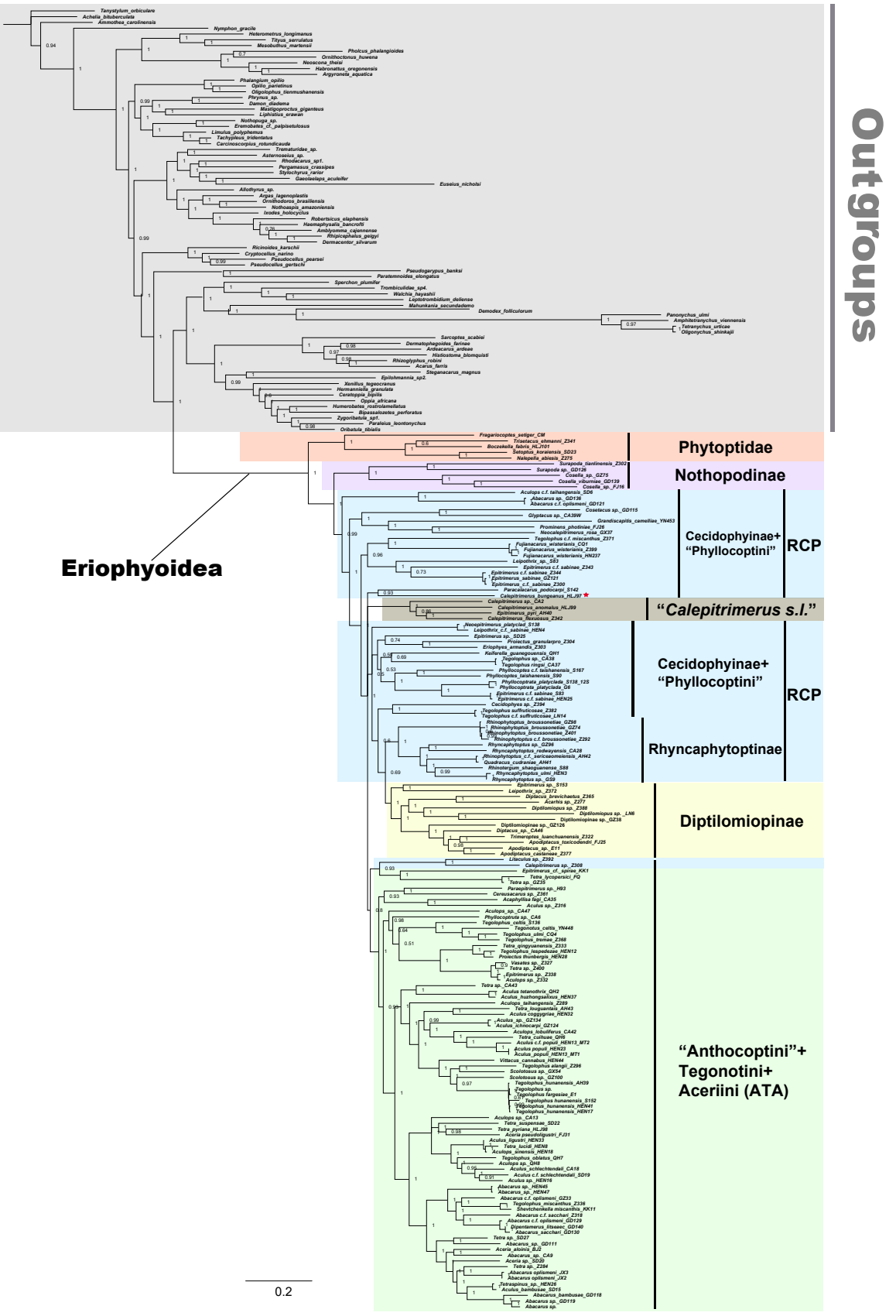

Additional file 2: Fig. S20. The phylogenetic tree inferred from mitochondrial amino acid sequences using PMSF method in IQ-TREE with LG + C20+F + G" model.

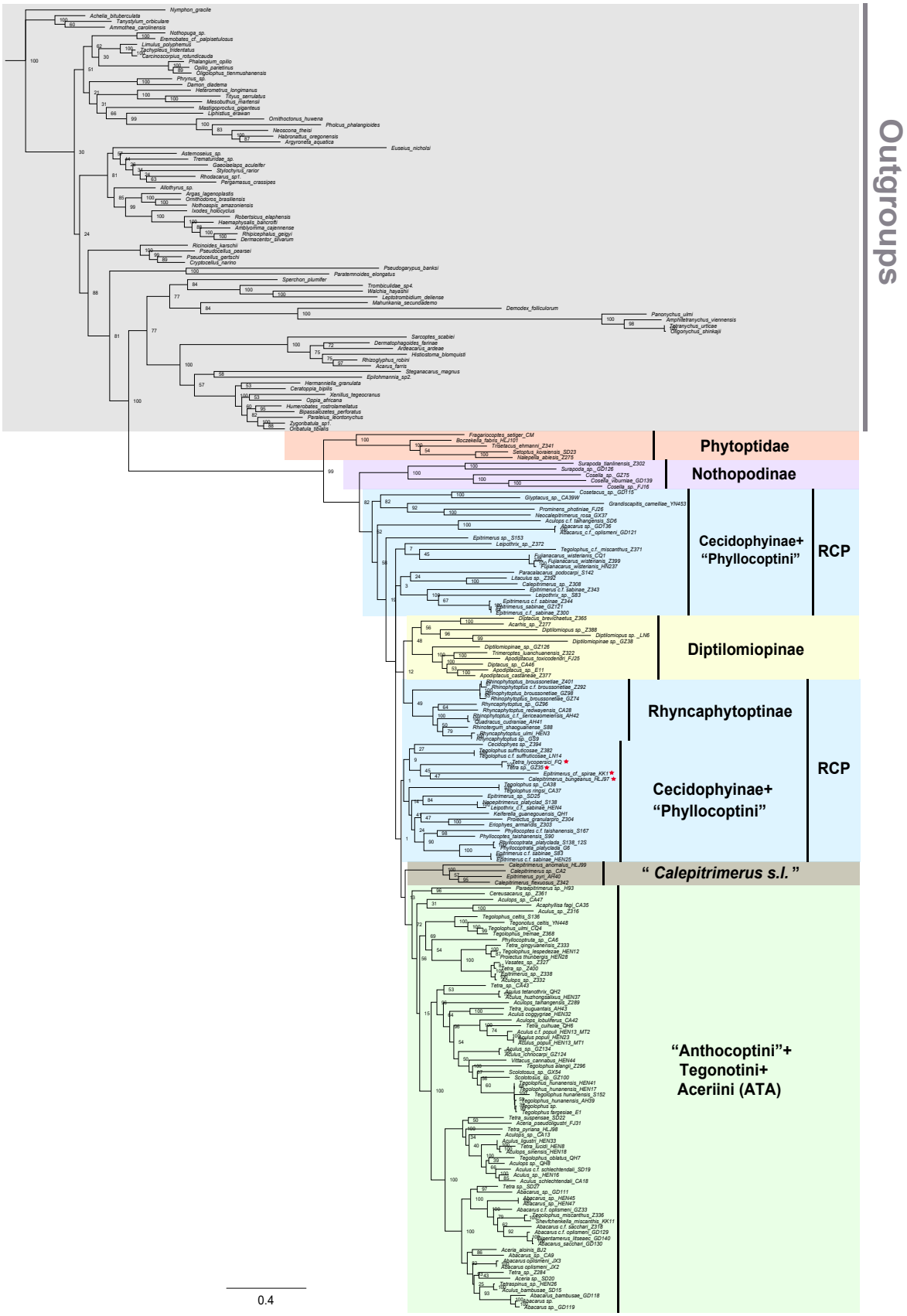

Additional file 2: Fig. S21. The relevant Navajo (sensitivity) plots to the 12 internal nodes of the RCP grade in Figure 2.

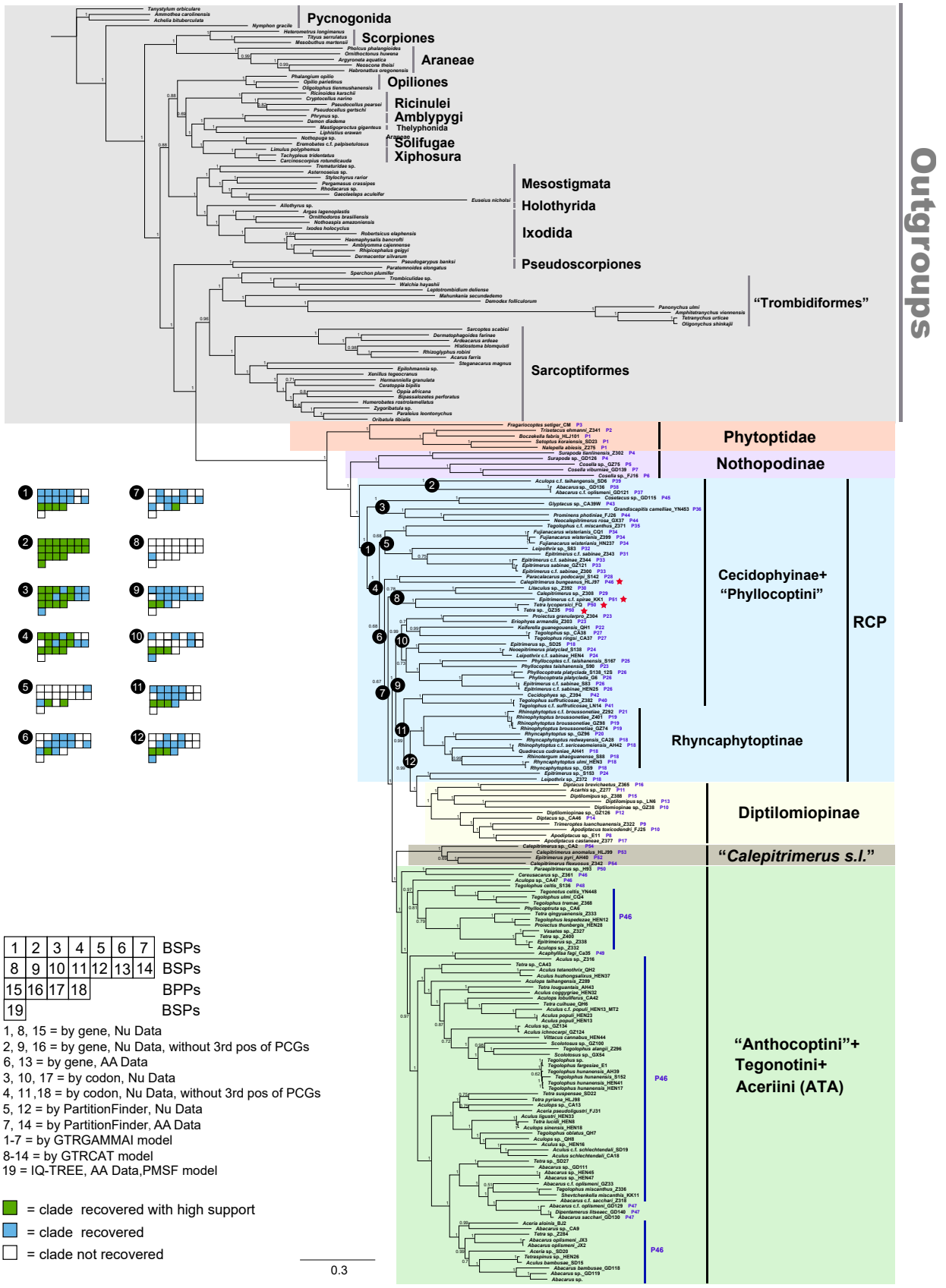

1	2	3	4	5	6	7	BSPs
8	9	10	11	12	13	14	BSPs
15	16	17	18				BPPs
19							BSPs

Additional file 2: Fig. S22. (a) Box plots of Ka (nonsynonymous substitutions per nonsynonymous site) in different arachnid lineages, marked by different colours. (b) Box plots of Bp (breakpoints) in different arachnid lineages, marked by different colours.

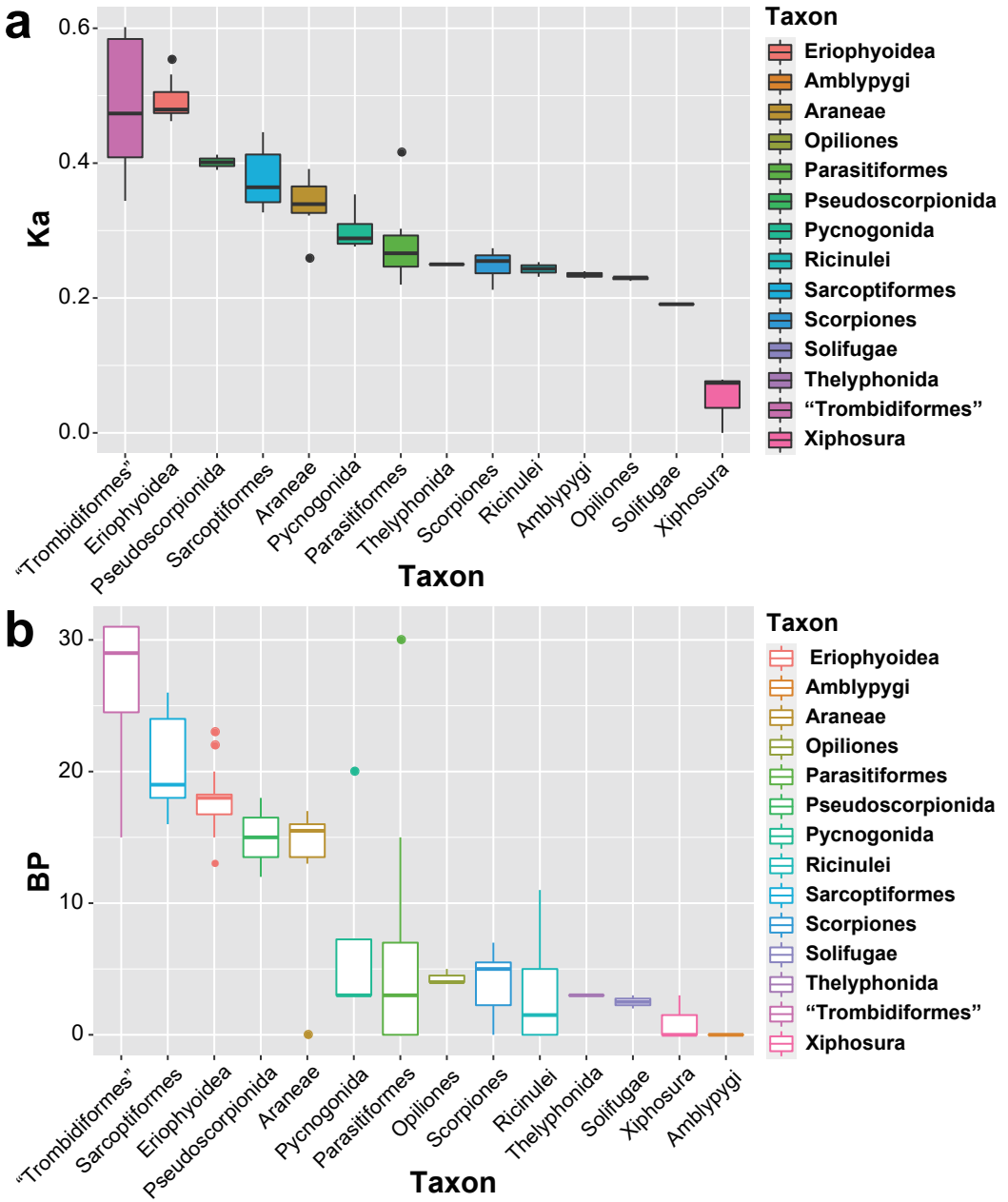

Supplement: Supplementary file 2 — Additional file 2: Fig. S1. The 54 mitochondrial gene arrangement patterns (Pattern 1–Pattern 54) from 153 eriophyoid mite species in this study. Genes underlined have opposite transcription orientation to those not underlined. Translocated or inverted genes are colour‐coded (blue: inversion and translocation; green: translocation; orange: inversion). Abbreviations of protein‐coding genes are atp6 and atp8 for ATP synthase subunits 6 and 8; cox1–3 for cytochrome oxidase subunits 1–3; cob for cytochrome b; nad1–6 and nad4L for NADH dehydrogenase subunits 1–6 and 4L; rrnL and rrnS for large and small rRNA subunits; tRNA genes are indicated by the single‐letter IUPAC‐IUB abbreviations for their corresponding amino acids. The shared rearranged mt gene clusters of each sample were denoted by underneath lines in different colors as the same as Fig. 1. Fig. S2. Mitochondrial gene arrangements of representative samples in the Eriophyoidea. Two samples were selected as representatives of each clade. Shared rearranged mt gene clusters of each sample were denoted by underneath lines in different colors. Underlined genes are encoded in the N‐strand. Translocated or inverted genes are colour-coded (blue: inversion and translocation; green: translocation; orange: inversion). Abbreviations of protein‐coding genes are atp6 and atp8 for ATP synthase subunits 6 and 8; cox1–3 for cytochrome oxidase subunits 1–3; cob for cytochrome b; nad1–6 and nad4L for NADH dehydrogenase subunits 1–6 and 4L; rrnL and rrnS for large and small rRNA subunits; tRNA genes are indicated by the single‐letter IUPAC‐IUB abbreviations for their corresponding amino acids.Fig. S3. The phylogenetic tree inferred from mitochondrial genome nucleotide sequences using maximum likelihood method with partition by gene and GTRGAMMAI model. Fig. S4. The phylogenetic tree inferred from mitochondrial genome nucleotide sequences (without the 3rd codon positions of PCGs) using maximum likelihood method with partition by gen [file 12915_2024_1870_MOESM2_ESM.pdf]
